# Self‐assembly of PEG–PPS polymers and LL‐37 peptide nanomicelles improves the oxidative microenvironment and promotes angiogenesis to facilitate chronic wound healing

**DOI:** 10.1002/btm2.10619

**Published:** 2023-11-08

**Authors:** Rong Shi, Jianxiong Qiao, Quanwu Sun, Biao Hou, Bo Li, Ji Zheng, Zhenzhen Zhang, Zhenxue Peng, Jing Zhou, Bingbing Shen, Jun Deng, Xuanfen Zhang

**Affiliations:** ^1^ Department of Plastic Surgery Lanzhou University Second Hospital Lanzhou Gansu China; ^2^ Department of Breast Surgery Gansu Provincial Hospital Lanzhou Gansu China; ^3^ Department of Joint Surgery and Sports Medicine Center for Orthopedic Surgery, Orthopedic Hospital of Guangdong Province, The Third Affiliated Hospital of Southern Medical University Guangzhou Guangdong China; ^4^ Department of Urology Xinqiao Hospital, Third Military Medical University (Army Medical University) Chongqing China; ^5^ Department of Nephrology Chongqing University Central Hospital, Chongqing Emergency Medical Center Chongqing China; ^6^ Institute of Burn Research, State Key Lab of Trauma, Burn, and Combined Injury, Chongqing Key Laboratory for Disease Proteomics Southwest Hospital, Third Military Medical University (Army Medical University) Chongqing China

**Keywords:** angiogenesis, diabetic wound healing, inflammatory microenvironment, LL‐37@PEG–PPS nanomicelles

## Abstract

Refractory diabetic wounds are associated with high incidence, mortality, and recurrence rates and are a devastating and rapidly growing clinical problem. However, treating these wounds is difficult owing to uncontrolled inflammatory microenvironments and defective angiogenesis in the affected areas, with no established effective treatment to the best of our knowledge. Herein, we optimized a dual functional therapeutic agent based on the assembly of LL‐37 peptides and diblock copolymer poly(ethylene glycol)–poly(propylene sulfide) (PEG–PPS). The incorporation of PEG–PPS enabled responsive or controlled LL‐37 peptide release in the presence of reactive oxygen species (ROS). LL‐37@PEG–PPS nanomicelles not only scavenged excessive ROS to improve the microenvironment for angiogenesis but also released LL‐37 peptides and protected them from degradation, thereby robustly increasing angiogenesis. Diabetic wounds treated with LL‐37@PEG–PPS exhibited accelerated and high‐quality wound healing in vivo. This study shows that LL‐37@PEG–PPS can restore beneficial angiogenesis in the wound microenvironment by continuously providing angiogenesis‐promoting signals. Thus, it may be a promising drug for improving chronic refractory wound healing.

**Translational Impact Statement**

Refractory diabetic wounds are a rapidly growing clinical concern, and their treatment is difficult due to the uncontrolled inflammatory microenvironment and defective angiogenesis. This study shows that LL‐37@PEG–PPS can restore and promote angiogenesis and facilitate epidermal cell migration by scavenging excessive reactive oxygen species and releasing LL‐37 peptides when necessary to confer protection against LL‐37 peptides degradation in the wound microenvironment, resulting in a robust wound healing. Thus, it may be a promising drug candidate to improve chronic refractory wound healing.

## 1 INTRODUCTION

Wound healing is one of the most complex but well‐orchestrated physiological process involving skin regeneration following injury.^1^ However, normal wound healing is impaired in conditions such as diabetes, infection, excessive skin pressure, and insufficient arterial or venous blood flow,^2^ resulting in chronic wounds.^3^ The conventional clinical treatment of chronic refractory diabetic wounds includes blood glucose control, surgery debridement, negative pressure therapy,^4^ antibiotics, and revascularization surgery, which mainly aim to prevent initial wound expansion.^5^ Although numerous advances have been made in treating nonhealing diabetic wounds, effectively treating chronic diabetic wounds remains challenging.^6^

Insufficient angiogenesis and local complex inflammatory microenvironments render it difficult for diabetic wounds to heal.^7^, ^8^ In chronic refractory diabetic wounds, the highly coordinated healing process is replaced by a persistent inflammatory state and long‐term microcirculation defects. Following tissue injury, the wound is in a persistent and high‐intensity inflammatory state because of the complex microenvironment of the wound, further hindering angiogenesis. The persistent and intensive inflammatory diabetic wound microenvironment reduces the healing capacity of epidermal cells and diminishes the formation of the collagen matrix,^9^, ^10^, ^11^ which need to be resolved for the appropriate healing of chronic wounds. Thus, regulating the inflammatory microenvironment and promoting angiogenesis are the key to diabetic wound healing.

LL‐37 belongs to the human cathelicin family and plays an important role in skin homeostasis. It exhibits antibacterial as well as anti‐inflammation and epidermal cell proliferation and migration regulatory properties. In addition, LL‐37 is produced by the innate immune system and has broad‐spectrum antibacterial properties.^12^ LL‐37 not only has microbicidal activity, it can activate epidermal growth factor receptor, induce cytokine release, and stimulate angiogenesis through G‐protein‐coupled receptor FPR2.^13^, ^14^, ^15^ Furthermore, LL‐37 functions as an effective calcium agonist on endothelial cells, inducing phosphorylation and cytoplasmic phospholipase A2 (cPLA2) activation, promoting PGE2 biosynthesis, and finally inducing prostaglandin‐dependent angiogenesis in vivo through the signal transduction of the PGE2 receptor EP3.^16^ Although LL‐37 has demonstrated great potential in promoting wound healing, its application is limited owing to its instability in the complex inflammatory microenvironment of diabetic wounds.^12^ Our previous studies have confirmed that nanomaterials possessing reactive oxygen species (ROS)‐scavenging properties improve the inflammatory microenvironment of wounds,^10^, ^17^, ^18^, ^19^ which is conducive to angiogenesis.^20^, ^21^ Therefore, constructing an LL‐37‐loaded nanocarrier with ROS‐scavenging ability may help address these problems.

**FIGURE 1:**
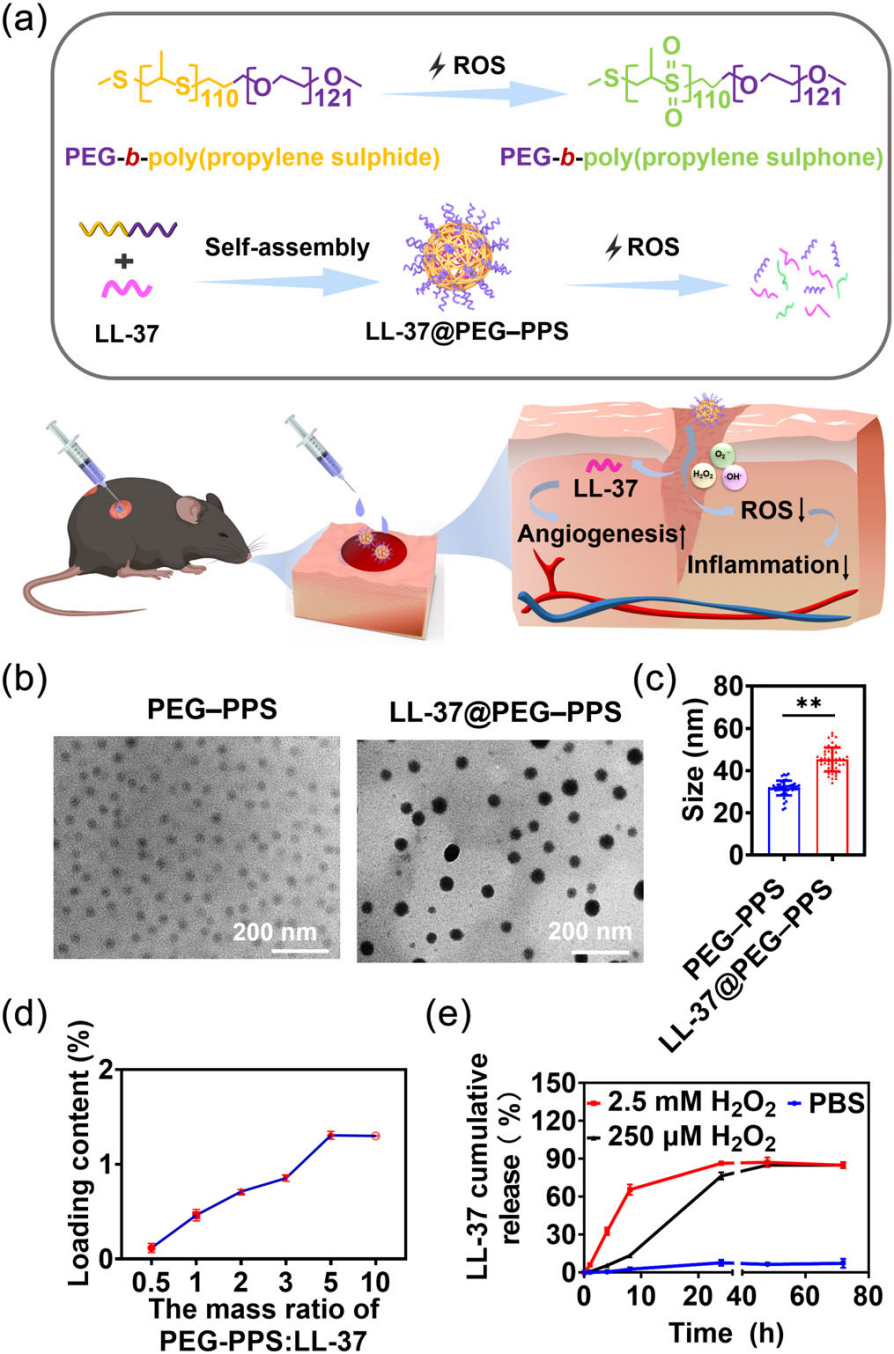
Synthesis diagram and characterization of nanomicelles. (a) The preparation of ROS‐responsive LL‐37@PEG–PPS in vitro and therapeutic mechanism of LL‐37@PEG–PPS in chronic wounds. (b) Representative TEM images of PEG–PPS and LL‐37@PEG–PPS nanomicelles. Scale bars: 200 nm. (c) The dry diameter of PEG–PPS and LL‐37@PEG–PPS nanomicelles was measured using Image J software. Data are expressed as mean ± SD (*n* = 50). *p < 0.05, **p < 0.01 (Student's *t*‐test). (d) The LC of LL‐37 in LL‐37@PEG–PPS nanomicelles was measured via HPLC (*n* = 3). (e) In vitro release rate curve of LL‐37 from LL‐37@PEG–PPS with 2.5 mM H_2_O_2_ and 250 μM H_2_O_2_ at predetermined timepoints (*n* = 3). HPLC, high‐performance liquid chromatography; LC, loading capacity; PEG–PPS, poly(ethylene glycol)–poly(propylene sulfide); ROS, reactive oxygen species; TEM, transmission electron microscope.

Herein, we developed an LL‐37‐loaded nanocarrier based on the assembly of poly(ethylene glycol)–poly(propylene sulfide) (PEG–PPS) diblock copolymers and LL‐37 polypeptide (LL‐37@PEG–PPS) (Figure 1a). PEG is a gold standard stealth polymer that can provide stable micelle dispersions. PPS can improve the stability of peptides and proteins in vivo, and thus exerts a protective effect on LL‐37.^22^ Each repeating unit of PPS contains a thioether group and can undergo oxidative conversion from the hydrophobic to hydrophilic surface, exerting a strong ROS‐scavenging ability and enabling on‐demand drug release.^23^ This nanomicelle platform can transform an unfavorable wound microenvironment into a favorable one by scavenging excessive ROS. In addition, controlled LL‐37 release induces the development of new blood vessels, thereby synergistically improving angiogenesis and accelerating diabetic wound healing. Herein, we demonstrated a simple but effective approach for vascular reconstruction in harsh oxidative microenvironments, which might contribute to the clinical delivery of growth factors for treating chronic diabetic wounds.

## 2 MATERIALS AND METHODS

### 2.1 Materials

PEG–PPS was obtained from Xian Ruixi Biological Technology Co., Ltd. (Xian, China). LL‐37 (LLGDFFRKSKEKIGKEFKRIVQRIKDFLRNLVPRTE) polypeptides and fluorescein isothiocyanate (FITC)‐labeled LL‐37 polypeptides were synthesized by Sangon Biotechnology Inc. (Shanghai, China). Dulbecco's modified Eagle's medium (DMEM), fetal bovine serum (FBS), penicillin–streptomycin solution, and trypsin were obtained from Gibco (Grand Island, NY, USA). Streptozotocin (STZ) and dihydroethidium (DHE, ≥95%) were acquired from Sigma‐Aldrich (St. Louis, MO, USA). Modified Masson's Trichrome Stain Kit (G1346) and Annexin V‐FITC Apoptosis Detection Kit (CA1020) were purchased from Beijing Solarbio Science & Technology Co., Ltd (Peking, China). 2′,7′‐Dichlorofluorescein diacetate (DCFH‐DA) and cell counting kit‐8 were purchased from MedChemExpress (MCE, USA). Human vascular endothelial growth factor (VEGF) enzyme‐linked immunosorbent assay (ELISA) kit (EK0539) was obtained from Boster Biological Technology Co., Ltd.

### 2.2 Animals

Adult C57BL/6 mice (male, 6–8 weeks old) were obtained from the Experimental Animal Center of the Third Military Medical University (Chongqing, China) (Animal Ethical Statement: AMUWEC20217007). The experimental animal program was approved by the Medical Animal Ethics Committee of the Third Military Medical University (Animal Ethical Statement: AMUWEC20217007). All animals were conventionally raised for 1 week before further experiments.

### 2.3 Cell culture

Human umbilical vein endothelial cells (HUVECs; ATCC CRL‐4053) were cultured in high‐glucose DMEM supplemented with 10% FBS and 1% penicillin–streptomycin. HaCaT cell line was purchased from the Chinese Academy of Sciences (Shanghai, China) and cultured in RPMI medium at 37°C under 5% CO_2_.

### 2.4 Preparation and characterization of LL‐37@PEG–PPS nanomicelles

LL‐37@PEG–PPS nanomicelles were prepared via the self‐assembly of PEG–PPS and LL‐37 polypeptides. LL‐37 is an amphiphilic cathepsin derived cathelicidin with 37 residues.^24^ PEG–PPS is an amphiphilic diblock copolymer characterized by a large quantity of sulfide structures on the main chain of PPS molecules, which constitutes the hydrophobic portion, while PEG constitutes the hydrophilic entity. Therefore, during the rapid stirring process, the hydrophobic forces aggregate the PPS and LL‐37 in the inner layer while the hydrophilic PEG tends to aggregate in the outer layer of the nanomicelles owing to hydrophilic forces, thereby loading LL‐37 peptides inside the nanomicelles. Briefly, 2 mg of LL‐37 polypeptides and 10 mg of PEG–PPS were mixed and dissolved in 1 mL tetrahydrofuran (THF) to which 10 mL deionized water was added and the solution was stirred for 1 h. Then, the solutions were centrifuged at 4°C for 30 min at 12,800 × *g*, the precipitate was collected, and the supernatant was discarded. The solvent was removed via repeated centrifugation, washing, and dialysis (molecular weight cutoff [MWCO] = 7 kDa) in deionized water at 4°C for 24 h to obtain LL‐37@PEG–PPS nanomicelles. FITC‐labeled LL‐37‐loaded PEG–PPS (FITC–LL‐37@PEG–PPS) nanomicelles were prepared via the same methods. PEG–PPS nanomicelles were fabricated using similar protocols without LL‐37.

Next, we prepared the oxidation products of PEG–PPS as follows: 2 mg PEG–PPS (*M*_w_ = 13,913) was mixed in 5 mL 2.5 mM H_2_O_2_ and stirred for 12 h at room temperature. After 24 h of dialysis (MWCO = 3.5 kDa), the reaction solution was lyophilized to obtain the oxidation products. Then, the oxidation products were characterized via Fourier‐transform infrared (FTIR) spectroscopy (Nicolet/Nexus 670 FTIR spectrometer, Thermo Fisher, USA) and ^1^H nuclear magnetic resonance (NMR, Bruker 400 MHz, Germany). The fluorescence and absorption spectra of FITC–LL‐37 were recorded via the microplate assay (Thermo Varioskan Flash, USA), with excitation and emission peaks of 488 and 530 nm, respectively.

### 2.5 Determination of the loading capacity and encapsulation efficiency of LL‐37

High‐performance liquid chromatography (HPLC, BRUKER AVANCE 400, Japan) was performed to measure the encapsulation efficiency (EF) and drug loading capacity (LC) of LL‐37 polypeptides. A transmission electron microscope (TEM, JEM‐1400plus, Japan) was used to determine the morphology and dry diameter of the LL‐37@PEG–PPS nanomicelles. A dynamic light scattering particle size analyzer (DLS, Zetasizer Nano ZSP, Malvern Instruments, UK) was used for measuring the zeta potential and hydrodynamic diameter of the nanomicelles.

HPLC (Ultimate 3000, ThermoFisher) was performed to measure the drug LC of the LL‐37 polypeptide. An LL‐37‐loaded micelle solution was first freeze‐dried, weighed, and redissolved in 2 mL dimethylsulfoxide (DMSO). The measurement was performed at 25°C using a Hypersil Gold‐C18 column. The mobile phases A and B comprised 0.1% trifluoroacetic acid–acetonitrile and 0.1% trifluoroacetic acid–water, respectively, delivered at a rate of 1.0 mL min^−1^. The injection volume was 30 μL and the detection wavelength was 214 nm. LL‐37 had a retention time of 8.19 min in the aforementioned experimental conditions. The concentration of LL‐37 was calculated based on the peak areas of the standard solutions (0.25, 1.25, 2.5, 4, 5, 35, 70, and 100 μg mL^−1^ in DMSO). The weight percentage of the freeze‐dried micelle was termed as the drug LC of LL‐37. The calibration curve of LL‐37 was constructed using the equation: concentration (μg mL^−1^) = 0.0937 × (*R* ^2^ = 0.999). The LC and EF of LL‐37 were calculated according to the following equation:

$$\mathrm{LC}\%=W_{1}/W\times 100\%,\;\mathrm{EF}\%=W_{1}/W_{2}\times 100\%,$$

where *W* is the mass of the LL‐37@PEG–PPS, *W*_1_ is the mass of LL‐37 in LL‐37@PEG–PPS, and *W*_2_ is the mass of the initial LL‐37 added.

### 2.6 In vitro drug release profile

Approximately 2 mg LL‐37@PEG–PPS nanomicelles (including 26 μg LL‐37) were dispersed in 500 μL phosphate‐buffered saline (PBS) and 500 μL 2.5 mM H_2_O_2_ and 250 μM H_2_O_2_ solution separately. Next, the solutions were centrifuged for 30 min at 12,800 × *g*. At 0, 1, 4, 8, 24, 48, and 72 h, 200 μL supernatant was removed from the solution and an equal amount of PBS was added. The amount of LL‐37 released at each timepoint was determined via HPLC. The injection volume was 30 μL and the content of LL‐37 was calculated based on the peak areas.

### 2.7 ROS‐scavenging ability of LL‐37@PEG–PPS nanomicelles

HUVECs were inoculated onto 12‐well plates at a density of 2 × 10^5^ cells per well and cultured overnight. Nonfluorescent DCFH is commonly used to measure cellular ROS level.^25^ First, the cells were cultured with 250 μM H_2_O_2_ for 4 h, following which different doses of LL‐37@PEG–PPS (containing 50–250 μg PEG–PPS and 0.66–3.30 μg LL‐37) and 250 μg PEG–PPS were administered to each group and cocultured for 24 h. Next, the cells were cocultured with DCFH‐DA for 15 min under dark conditions. Finally, the cells were washed with PBS to remove unloaded probes. The intracellular fluorescence intensity of the cells was detected via flow cytometry.

To evaluate the antiapoptotic ability of the LL‐37@PEG–PPS nanomicelles, Annexin V/propidium (PI) apoptosis kit was used. HUVECs were inoculated onto 12‐well plates and cultured overnight and then treated with 250 μM H_2_O_2_ and LL‐37@PEG–PPS at the indicated concentrations (50–250 μg PEG–PPS and 0.66–3.3 μg LL‐37) for another 24 h. Next, the cells were harvested and resuspended. Then, the cells were incubated with Annexin V‐FITC (5 μL) and PI (10 μL) under dark conditions for 15 min and subsequently visualized using flow cytometry. Data were analyzed using FlowJo software (Tree Star, USA). Each experiment was repeated at least thrice.

### 2.8 In vitro tube‐formation assay

Tube‐formation assays were performed according to our previous report.^26^ Matrigel (BD, USA) was diluted with DMEM at a proportion of 1:1. Then, each well of an angiogenesis slide (Ibidi, Germany) was coated with 10 μL diluted Matrigel. After 30 min, 1 × 10^4^ HUVECs treated with 250 μM H_2_O_2_ for 4 h were seeded into each well and cocultured with 100 μL PBS, 200 μg PEG–PPS, and 2.6 μg LL‐37 and LL‐37@PEG–PPS (containing 2.6 μg LL‐37 and 197 μg PEG–PPS) for 4 h. Finally, the tube formation was photographed. The number of tubes and nodes and the total length of the tubes were measured using Image J software (NIH, USA). Each experiment was repeated at least thrice.

### 2.9 In vitro scratch‐wound and Transwell assays

Scratch‐wound assay was performed to measure the cell migration speed. Approximately 2 × 10^5^ HUVECs and 2 × 10^5^ HaCaT cells were inoculated onto 12‐well plates and cultured to 90% confluency. First, the cells were cocultured with 250 μM H_2_O_2_ for 4 h, and a straight‐line scratch was created using a 200‐μL pipette tip. Then, each group was subjected to different treatments, and photographs were collected under a microscope at predetermined timepoints.

A 12‐well Transwell chamber (Corning, USA) with 8‐μm pore size was used to observe the migration of HUVECs. Approximately 3 × 10^5^ cells were inoculated onto the upper chamber containing serum‐free DMEM, and DMEM containing 10% FBS was used in the lower chamber; the cells were treated with H_2_O_2_. Next, 100 μL PBS, 200 μg PEG–PPS, and 2.6 μg LL‐37 and LL‐37@PEG–PPS (containing 2.6 μg LL‐37 and 197 μg PEG–PPS) were added into the lower chamber and then cocultured for 24 h. The cells that passed through the pores were fixed with methanol, stained with 1% crystal violet, and enumerated.

### 2.10 In vitro and in vivo biocompatibility of the LL‐37@PEG–PPS nanomicelles

Approximately 1 × 10^4^ HUVECs were incubated onto 96‐well plates for 24 h. LL‐37@PEG–PPS at various concentrations was added to the confluent monolayer of HUVECs, which was formed after 24 h. Then, the optical density (OD) was recorded at 450 nm.

Fresh blood was collected from the orbital vein of male Sprague–Dawley rats and centrifuged at 1500 × *g* for 15 min. The precipitated red blood cells were washed several times with normal saline until the supernatant was no longer red. Then, LL‐37@PEG–PPS (1 mL) was added to 100 μL diluted red blood cells at different concentrations (100–10,000 μg mL^−1^). Following incubation at room temperature for a certain period, the OD of the sample supernatant (100 μL) was measured at 540 nm.

To further examine the toxicity of LL‐37@PEP–PPS in vivo, 2 mg kg^−1^ LL‐37@PEG–PPS (10‐fold higher than that required for treating diabetic wounds) was intravenously administered every 3 days. The control group was treated with normal saline. The whole blood was harvested, and the animals were euthanized via cervical dislocation on day 12. The heart, liver, spleen, lungs, and kidneys were harvested for hematoxylin and eosin (H&E) staining. Routine serum examination and serum biochemistry tests, such as liver and kidney function tests, were performed. The liver function test indices included alanine aminotransferase and aspartate aminotransferase levels, and the kidney function test indices included creatinine and blood urea nitrogen levels.

### 2.11 In vivo distribution of LL‐37@PEG–PPS

C57BL/C mice (male, 8–10 weeks old) were used to establish an induced diabetes model. A type I diabetic mouse model was established using STZ (100 mg kg^−1^) according to a previously reported method.^18^ Only mice with serum glucose levels of >16.7 mM were considered diabetic mice.^17^

Diabetic mice were used to observe the biological distribution of LL‐37@PEG–PPS. The mice were anesthetized using 1% pentobarbital sodium (50 mg kg^−1^) and their backs were depilated to reduce background signals. FITC–LL‐37@PEG–PPS (200 μg) and PBS (100 μL) were subcutaneously injected into the back of the mice. In vitro optical imaging (AniView 100, China) was conducted to determine the distribution of FITC–LL‐37 at a predetermined timepoint. The accumulation of FITC–LL‐37 in different organs was evaluated by euthanizing the mice via cervical dislocation on day 4 following injection, after which the main organs (heart, liver, spleen, lungs, and kidneys) were harvested for in vitro optical imaging. Finally, the average fluorescence intensity was recorded using AniView 100 software.

### 2.12 Therapeutic effects of LL‐37@PEG–PPS on a diabetic wound model

Type I diabetic mice were used for the wound experiments. After administering anesthesia with 1% pentobarbital sodium, a round full‐thickness skin defect wound with a diameter of 6 mm was created, and a green card (with a diameter of 6 mm) of the same size and shape was used as a reference ruler. The mice were randomly divided into four groups and administered with 100 μL PBS, 200 μg PEG–PPS, and 2.6 μg LL‐37 and LL‐37@PEG–PPS (containing 2.6 μg LL‐37 and 197 μg PEG–PPS). Then, a transparent film (Tegaderm film, USA) was applied on each wound surface to prevent wound contraction to some extent and drug overflow. The wounds were photographed using a digital camera at a predetermined timepoint. The following formula was used to calculate the wound closure rate:

$$\text{Wound closure rate}\;\left(\%\right)=\left(A_{I}-A_{R}\right)/A_{I}\times 100,$$

where *A*_I_ represents the initial wound area and *A*_R_ indicates the remaining wound area after healing at a predetermined timepoint.

### 2.13 Histological examination

Wound tissues were fixed in 4% paraformaldehyde at each timepoint. These tissues were subsequently dehydrated, embedded into transparent wax, and sectioned into 6‐mm blocks. The histopathological experiment was double blind and conducted by a pathologist.

#### 2.13.1 H&E staining

The tissue sections were dewaxed in xylene, dehydrated in ethanol, and then sealed using neutral resin and cover slips. The H&E‐stained sections were used to determine the thickness of the granulation tissue and length of the new epidermis.

#### 2.13.2 Masson's trichrome staining

Staining was performed using Masson's modified trichrome staining kit (Solarbio, China) according to the manufacturer's instructions. These tissue sections were stained using Masson's compound staining and aniline blue solutions, washed with absolute ethanol, and dried naturally. The following formula was used to calculate the wound collagen volume fraction:

$$\text{Collagen volume fraction}\;\left(\%\right)=A_{B}/A_{T}\times 100,$$

where *A*_B_ represents the collagen positive blue area and *A*_T_ indicates the total tissue area after healing at a predetermined timepoint.

#### 2.13.3 Immunohistochemistry and immunofluorescence staining

After first incubating the tissue sections with primary antibodies (rabbit anti‐mouse CD31 [1:50, ABclonal, Cat# A0378], anti‐interleukin (IL)‐1 beta rabbit pAb [1:800, Servicebio, GB11113‐100], and tumor necrosis factor (TNF) Alpha [1:400, Proteintech, Cat No. 60291‐1‐lg]) at 4°C for 24 h, the sections were incubated with biotin‐labeled secondary antibody and horseradish enzyme‐labeled streptomyces at room temperature for 2 h. The color reaction was performed using diaminobenzidine (DAB) kit. Second, the tissue sections were incubated with rabbit anti‐mouse CD31 (1:50, ABclonal, Cat# A0378) primary antibody at 4°C for 24 h, following which they were incubated with goat anti‐rabbit IgG (Alexa Fluor®647, 1:500, Abcam, Cat# ab150083) secondary antibody at room temperature for 2 h. Finally, all the tissue sections were scanned using a full slide scanner (SLIDEVIEW VS200; Olympus) and all the images were analyzed using OlyVIA software.

### 2.14 Statistical analysis

Statistical mean differences were analyzed using GraphPad Software 8.0 (GraphPad Software, USA). Student's *t*‐test and unpaired *t*‐test were performed to compare the significant differences between two groups, whereas two or more groups were analyzed using analysis of variance. Tukey's test was used when differences were detected. Data in each group are expressed as mean ± SD (*p < 0.05, **p < 0.01). Bliss independence (BLISS) was performed for the evaluation of the synergy scores.

## 3 RESULTS AND DISCUSSION

### 3.1 Characterization of LL‐37@PEG‐PPS

The morphology of blank PEG–PPS and LL‐37@PEG–PPS nanomicelles was observed via TEM, which showed that both PEG–PPS and LL‐37@PEG–PPS nanomicelles were spherical. The average dry diameters of PEG–PPS and LL‐37@ PEG–PPS nanomicelles were 32 ± 4 and 45 ± 8 nm, respectively (Figure 1b,c). The hydrodynamic diameters of PEG–PPS and LL‐37@PEG–PPS nanomicelles were 91 and 122 nm, respectively (Figure S1). The particle distribution indices of PEG–PPS and LL‐37@PEG–PPS were 0.177 and 0.198, respectively. The zeta potentials of PEG–PPS and LL‐37@PEG–PPS in PBS at pH 7.0 were −2.01 and 24.8 mV, respectively (Figure S2). ^1^H NMR (400 MHz, DMSO‐*d*_6_) *δ* 3.48–3.52 (m, b + c), 2.92–3.01 (m, a), 2.91–2.85 (m, d), 2.69–2.59 (m, f), 1.25–1.31 (m, e), and 1.23 (s, SH) (Figure S3) was performed for characterizing the PEG–PPS nanomicelles. We attempted to increase the LC of LL‐37 by adjusting the mass ratio between LL‐37 and PEG–PPS, as the LC of LL‐37 is known to reach its maximum when the mass ratio of PEG–PPS to LL‐37 is 5:1. The maximum LC of LL‐37 in LL‐37@PEG–PPS nanomicelles was 1.3% (Figure 1d) and the EF of LL‐37 was 14.2 ± 2.3% as determined via HPLC (Figure S4).

The accumulative release rate of LL‐37 from LL‐37@PEG–PPS was measured for 3 days in vitro via HPLC. At 4 h, 32 ± 3% LL‐37 was detected in the supernatant of the 2.5 mM H_2_O_2_ solution. At 8 h, 65 ± 4% LL‐37 was detected in the supernatant portion of the 250 μM H_2_O_2_ solution. At 24 h, 86 ± 1.7% and 76 ± 2.8% LL‐37 was detected in the supernatant portion of the 2.5 mM and 250 μM H_2_O_2_ solution, respectively, indicating that LL‐37 was initially burst‐released and the subsequent release lasted until approximately day 3 (Figure S5). The release of LL‐37 from LL‐37@PEG–PPS in PBS was relatively slow, with a cumulative release rate of 7.07 ± 2.9% (Figure 1e).

Furthermore, we evaluated the secondary protein structure of free LL‐37, loaded LL‐37, and LL‐37 released from LL‐37@PEG–PPS via circular dichroism (CD) analysis by scanning LL‐37 in the wavelength range of 190–260 nm. From the CD spectra obtained (Figure S6), the protein secondary structure in the form of α‐helix, β‐sheet, β‐turn, and random coil percentages was determined using CD spectral analysis software (CDPro, calculation program Slecon3). As evident from Table 1, the protein secondary structure proportions of free LL‐37, loaded LL‐37, and LL‐37 released from LL‐37@PEG–PPS were almost the same, indicating that PEG–PPS can effectively protect LL‐37 from degradation.

**TABLE 1:** Proportion of protein secondary structure of free LL‐37, loading LL‐37, and released LL‐37.

	α‐helix	β‐sheet	β‐turn	Random coils
Free LL‐37	40%	39%	21%	33%
Loading LL‐37	38%	40%	23%	34%
Released LL‐37	39%	39%	22%	36%

### 3.2 ROS‐responsive release of LL‐37 in vitro

**FIGURE 2:**
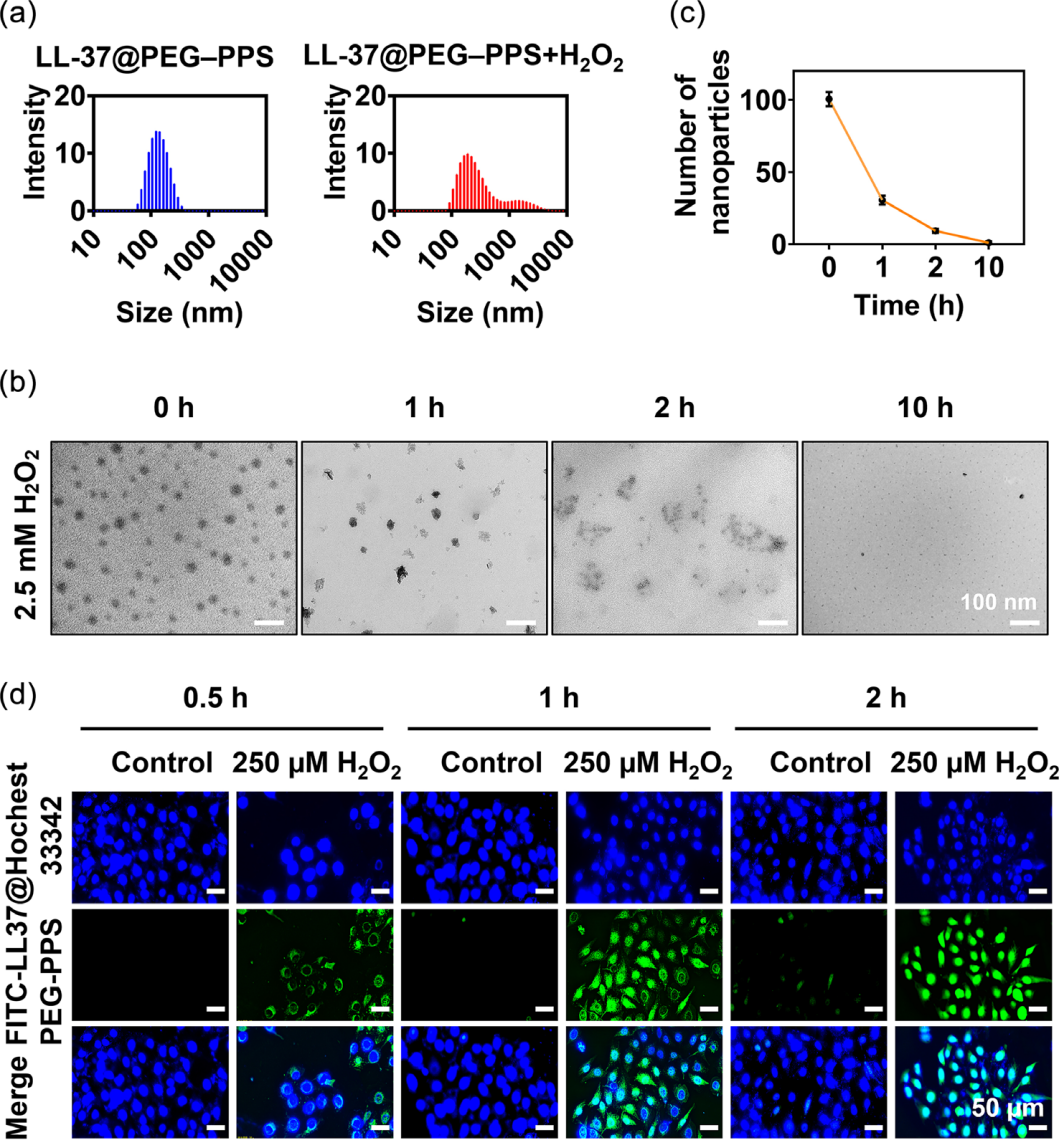
ROS responsiveness of LL‐37@PEG–PPS nanomicelles. (a) The hydrodynamic diameter distribution of LL‐37@PEG–PPS nanomicelles in PBS solution and 2.5 mM H_2_O_2_ was measured using DLS. (b) TEM images of LL‐37@PEG–PPS nanomicelles following treatment with 2.5 mM H_2_O_2_ for 0, 1, 2, and 10 h. Scale bar: 100 nm. (c) The number of LL‐37@PEG–PPS nanomicelles with a dry diameter of ≥28 nm were counted at different timepoints following treatment with 2.5 mM H_2_O_2_ (*n* = 3). (d) After coculture with FITC@LL‐37@PEG–PPS for 0.5, 1, and 2 h, confocal microscopy was performed to assess the amount of released FITC@LL‐37 in HUVECs. Scale bar: 50 μm (*n* = 3). DLS, dynamic light scattering; FITC, fluorescein isothiocyanate; HUVECs, human umbilical vein endothelial cells; PBS, phosphate‐buffered saline; PEG–PPS, poly(ethylene glycol)–poly(propylene sulfide); ROS, reactive oxygen species; TEM, transmission electron microscope.

To assess the oxidation sensitivity of LL‐37@PEG–PPS toward H_2_O_2_, we treated PEG–PPS with 2.5 mM H_2_O_2_ for 10 h and measured its particle size(Figure 2a). The average particle size of LL‐37@PEG–PPS in 2.5 mM H_2_O_2_ considerably increased from 122 ± 46 to 413 ± 362 nm within 2 h (Figure 2b). Because the H_2_O_2_ concentration is between 100 and 250 μM in skin wounds,^27^, ^28^ we incubated LL‐37@PEG–PPS with H_2_O_2_ (250 μM, 2.5 mM) at room temperature to observe the ROS response of LL‐37@PEG–PPS at the scheduled timepoint. The solution was characterized via TEM, and nanoparticles with a diameter of ≥28 nm were counted. The TEM images indicated that the shells of the micelles gradually dissolved (Figure S7) following incubation with 250 μM H_2_O_2_ for only 1 h. In addition, the TEM images indicated that following incubation with 2.5 mM H_2_O_2_ for only 1 h, the morphology of the nanomicelles changed from spherical to irregular (Figure 2c). After 10 h, the number of nanoparticles with a diameter of ≥28 nm significantly decreased. These results were largely attributed to hydrophobic sulfides that transformed into hydrophilic sulfoxides and sulfones, leading to the swelling and disassembly of the nanomicelles. The oxidative products of LL‐37@PEG–PPS nanomicelles were characterized via ^1^H NMR: ^1^H NMR (400 MHz, THF‐*d*_6_) *δ* 4.09–3.97 (m, b), 3.97–3.88 (m, c), 3.86–3.74 (m, a), 3.69–3.58 (m, d), 3.46–3.39 (m, f), 1.60–1.46 (m, e), and 1.43 (m, SH) (Figure S8). The LL‐37@PEG–PPS nanomicelles before and after their reaction with ROS were characterized via FTIR spectroscopy. A peak was observed at 1022 cm^−1^ (Figure S9). FTIR indicated a predominant sulfone presence (νs. SO_2_) and symmetric (νs. SO_2_) stretching bands of sulfones at ~1300 and 1120 cm^−1^. This result indicated that LL‐37@PEG‐PPS nanomicelles exhibited good ROS‐responsive release ability.

HUVECs were selected to further assess the LL‐37‐release capability of the nanomicelles at the cellular level. FITC–LL‐37@PEG–PPS was observed in cells treated with 250 μM H_2_O_2_ after coculture for only 30 min (Figure 2d). The fluorescence intensity of intracellular FITC–LL‐37@PEG–PPS gradually increased and accumulated in the cells for at least 2 h, indicating that FITC–LL‐37@PEG–PPS disassembled following the action of intracellular ROS, releasing FITC–LL‐37. The released FITC–LL‐37 gradually entered the nucleus. However, compared with the cells without H_2_O_2_ treatment, the fluorescence intensity of intracellular FITC–LL‐37@PEG–PPS barely changed (Figure S10).

### 3.3 In vitro scavenging of cellular ROS

**FIGURE 3:**
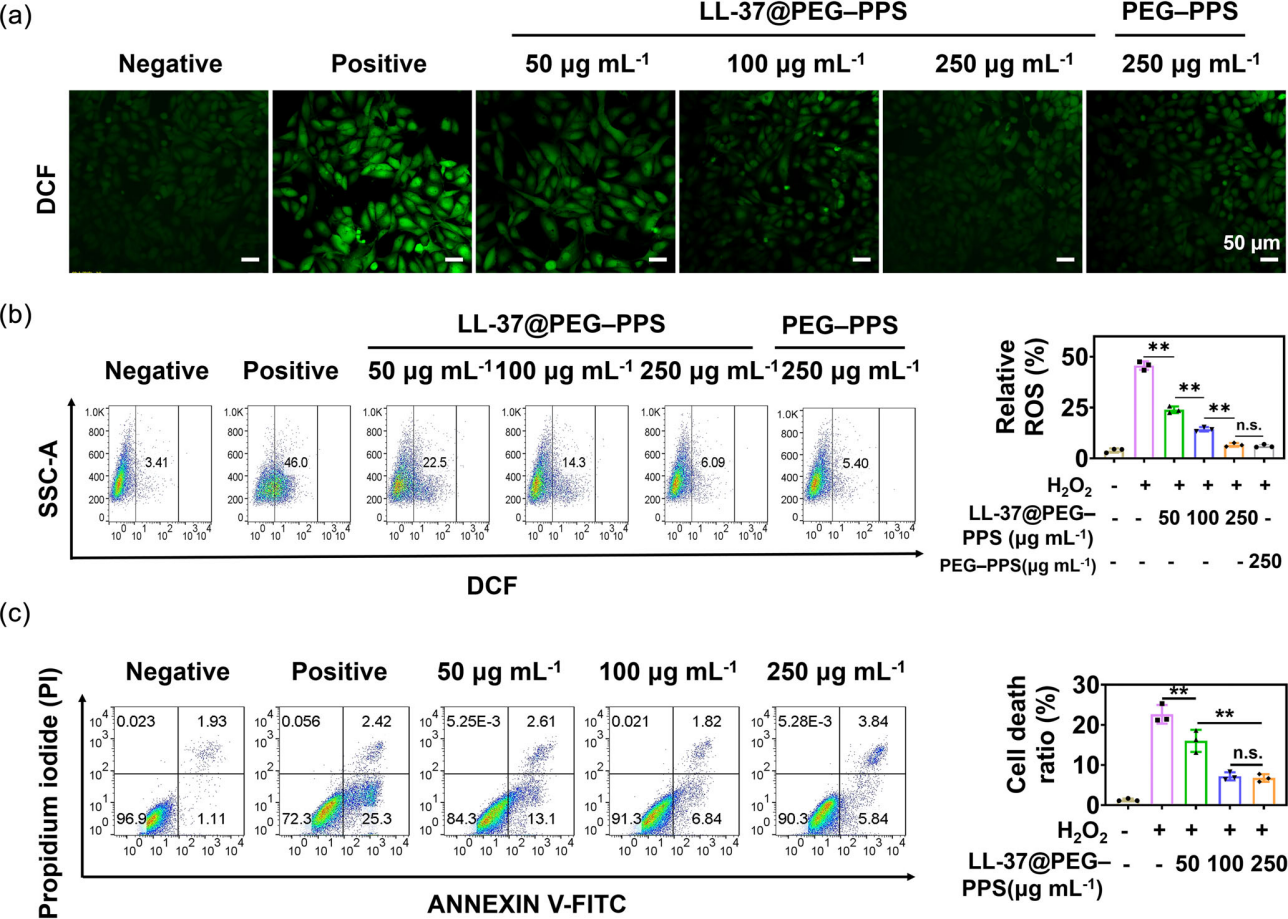
Alleviating effect of LL‐37@PEG‐PPS on reactive oxygen species and apoptosis in vitro. (a) Reactive oxygen species (ROS) level in untreated and LL‐37@PEG–PPS‐treated HUVECs was quantified following incubation with 250 μM H_2_O_2_. Scale bar = 50 μm. (b) ROS levels in HUVECs under different treatments were analyzed via flow cytometry (*n* = 3). (c) Cell death (both apoptosis and necrosis) under different treatment conditions was assessed via flow cytometry. Data in each group are expressed as mean ± SD (*n* = 3). *p < 0.05, **p < 0.01, n.s., no significance (one‐way ANOVA). HUVECs, human umbilical vein endothelial cells; PEG–PPS, poly(ethylene glycol)–poly(propylene sulfide).

Cellular ROS oxidizes DCFH (which emits no fluorescence) to DCFH‐DA (which emits fluorescence).^29^, ^30^ The intracellular ROS level is correlated with the fluorescence intensity of 2′,7′‐dichlorofluorescein, the oxidation product of DCFH. Thus, DCFH‐DA serves as a fluorescent probe for detecting the ROS level in HUVECs. The intracellular ROS levels (green fluorescent signal) in the HUVECs significantly increased following treatment with 250 μM H_2_O_2_ (Figure 3a). Conversely, the intracellular ROS levels significantly reduced following treatment with 50 μg mL^−1^ LL‐37@PEG–PPS. The level of ROS in HUVECs decreased with increasing concentrations of LL‐37@PEG–PPS. The intracellular ROS levels of the HUVECs decreased to the same level as those in the control group when the concentrations of LL‐37@PEG–PPS and PEG–PPS were 250 μg mL^−1^, thus demonstrating good ROS‐scavenging ability (Figure S11). The changes in the intracellular ROS levels were subsequently quantitatively analyzed via flow cytometry and the results further confirmed this trend. The ROS level in the negative control group (without H_2_O_2_) was 3.41%, whereas that in HUVECs significantly increased to 46% following treatment with 250 μM H_2_O_2_. However, after pretreatment with various concentrations of LL‐37@PEG–PPS, the intracellular ROS level decreased. At a PEG–PPS concentration of 250 μg mL^−1^, the intracellular ROS level approximately decreased to that of the negative control group (without H_2_O_2_) (Figure 3b).

Additionally, the antiapoptotic ability of LL‐37@PEG–PPS in H_2_O_2_‐treated cells was determined via flow cytometry. LL‐37@PEG–PPS played an antiapoptotic role in HUVECs induced by H_2_O_2_ (Figure 3c; *p* < 0.01). The results demonstrate the ROS‐scavenging ability of LL‐37@PEG–PPS and its protective effect on cells.

### 3.4 Tube‐formation and migration assays

**FIGURE 4:**
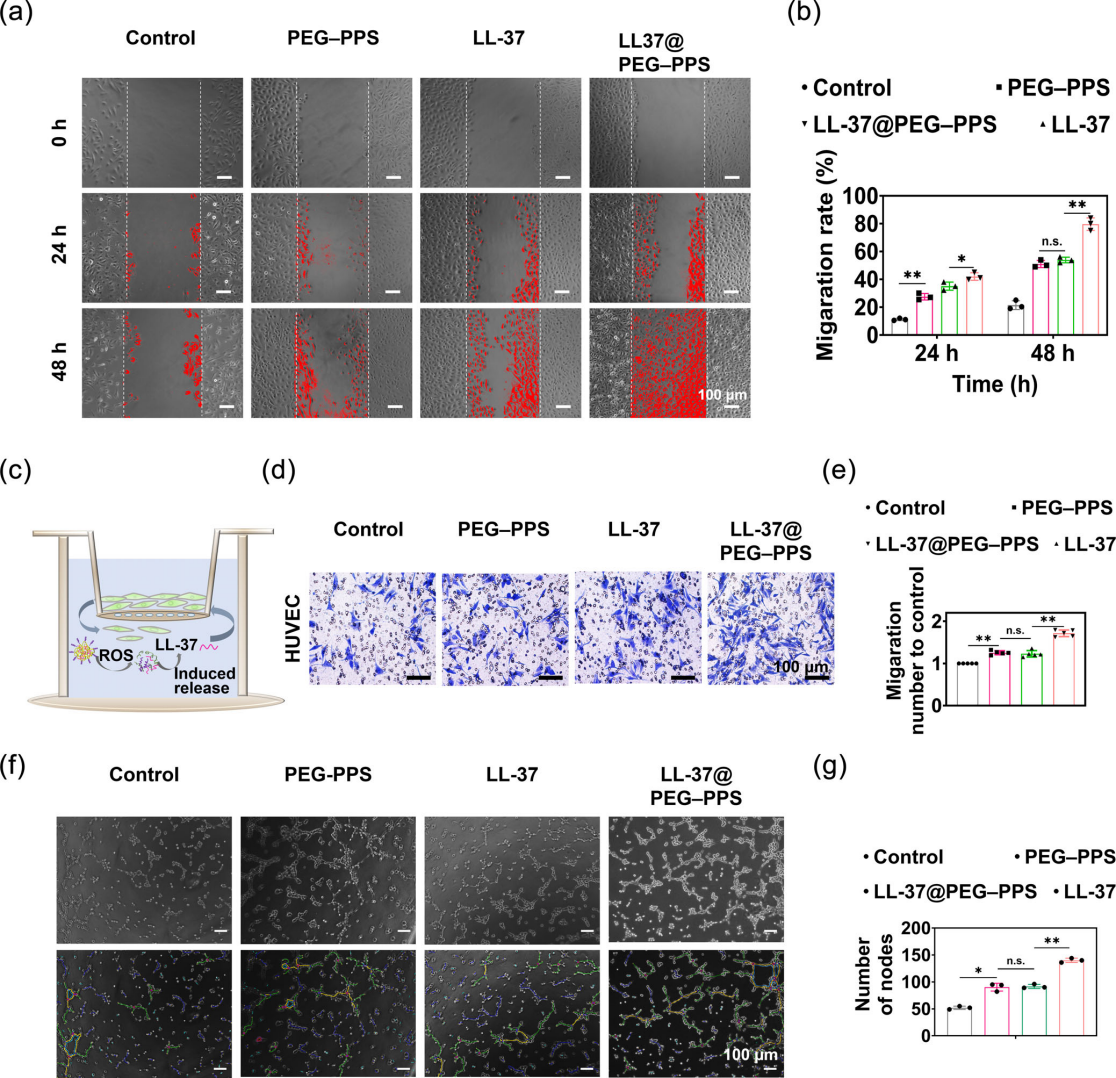
Promoting effect of LL‐37@PEG–PPS on cell migration and angiogenesis. (a) Representative images of scratch tests for HUVECs under PBS, LL‐37, PEG–PPS, and LL‐37@PEG–PPS treatments and (b) quantitative analysis of cell migration (*n* = 3). (c) Experimental schematic diagram of the migration effect of LL‐37@PEG–PPS on HUVECs. (d) Representative crystal violet staining images of HUVEC migration under different treatments via Transwell assays and (e) quantitative analysis (*n* = 5). (f) The angiogenic effects of PEG–PPS, LL‐37, and LL‐37@PEG–PPS on HUVECs. (g) Quantitative analysis of the number of nodes. Data are represented as mean ± SD (*n* = 3). *p < 0.05, **p < 0.01, n.s., no significance, (one‐way ANOVA). HUVECs, human umbilical vein endothelial cells; PEG–PPS, poly(ethylene glycol)–poly(propylene sulfide).

Previous studies have confirmed that LL‐37 promotes the migration of endothelial cells involved in angiogenesis.^31^, ^32^, ^33^ In addition, the site‐specific migration of keratinocytes plays an important role in wound healing.^34^ HUVECs and HaCaT cells were used to evaluate the effect of LL‐37@PEG–PPS on the migration ability of vascular endothelium cells and keratinocytes, respectively (Figure 4a and Figure S12A). Cell scratch test was performed to verify the ability of LL‐37@PEG–PPS to promote cell migration following preincubation with 250 μM H_2_O_2_ for 4 h. LL‐37@PEG–PPS exhibtied remarkable migration ability in vascular endothelium cells and keratinocytes (Figure 4b and Figure S12B). Although both LL‐37 and PEG–PPS treatments promoted cell migration, LL‐37@PEG–PPS treatment demonstrated a stronger effect to promote cell migration, which may be attributed to the combination of LL‐37 and PEG–PPS (*p* < 0.01). In addition, Transwell chamber tests were performed to verify the ability of LL‐37@PEG–PPS to promote vascular endothelium cell migration (Figure 4c). Both LL‐37 and PEG–PPS treatments exhibtied a good ability to promote HUVEC migration compared with the control group (*p* < 0.01) (Figure 4d,e). The cell migration–promoting effect observed in the PEG–PPS group may be because of the ROS‐scavenging and indirect cell migration–promoting roles of PEG–PPS.

Microvascular endothelium in the dermis is crucial for angiogenesis during skin wound healing.^35^ HUVECs treated with 250 μM H_2_O_2_ were used to evaluate the effect of LL‐37@PEG–PPS on angiogenesis. The LL‐37@PEG–PPS group exhibited enhanced tube formation than the other treatment groups (Figure 4f,g and Figure S13A,B). In addition, the PEG–PPS group demonstrated good tube‐formation ability compared with the control group (*p* < 0.01), which can be attributed to the ROS‐scavenging ability of PEG–PPS that helps create a conducive microenvironment to promote vascular regeneration. VEGF plays an important role in angiogenesis.^36^ Therefore, we evaluated the effects of different treatments on the VEGF‐secreting ability of HUVECs. After 48 h of different treatments on HUVECs, the cell supernatants were quantitatively analyzed for VEGF levels via the ELISA assay. The trend of the results was consistent with that in Figure 4 and Figure S14. Thus, the positive effect of LL‐37@PEG–PPS on angiogenesis was further confirmed.

### 3.5 Biodistribution of LL‐37@PEG–PPS in diabetic mice following local administration

To observe the distribution of LL‐37@PEG–PPS in diabetic mice, FITC–LL‐37 was self‐assembled with PEG–PPS to form nanomicelles for in vivo fluorescence imaging. Following the subcutaneous administration of FITC–LL‐37@PEG–PPS into the back of the mice, the fluorescence signals of FITC–LL‐37 were traced at 0 min, 5 min, 12 h, 1 day, 2 days, 3 days, and 4 days, respectively. The fluorescence signal of FITC–LL‐37 in the subcutaneous tissues of the mice lasted for >3 days, becoming considerably weak on day 4 (Figure S15A). Therefore, these results indicate that local drugs should be administered 0, 3, and 6 days after surgery to maintain effective LL‐37 levels on the wound surface, thereby providing sufficient time for early chronic wounds to induce vascular endothelial cell migration and angiogenesis in the early stage of wound healing. On day 4, in vitro fluorescence signal analysis was reconducted on the major organs, viz. the heart, liver, spleen, lungs, and kidneys, of the mice from the PBS and FITC–LL‐37@PEG–PPS groups; no signals were recorded from these organs (Figure S15B), indicating that FITC–LL‐37@PEG–PPS can be safely metabolized.

### 3.6 Therapeutic effect of LL‐37@PEG–PPS in diabetic mice

**FIGURE 5:**
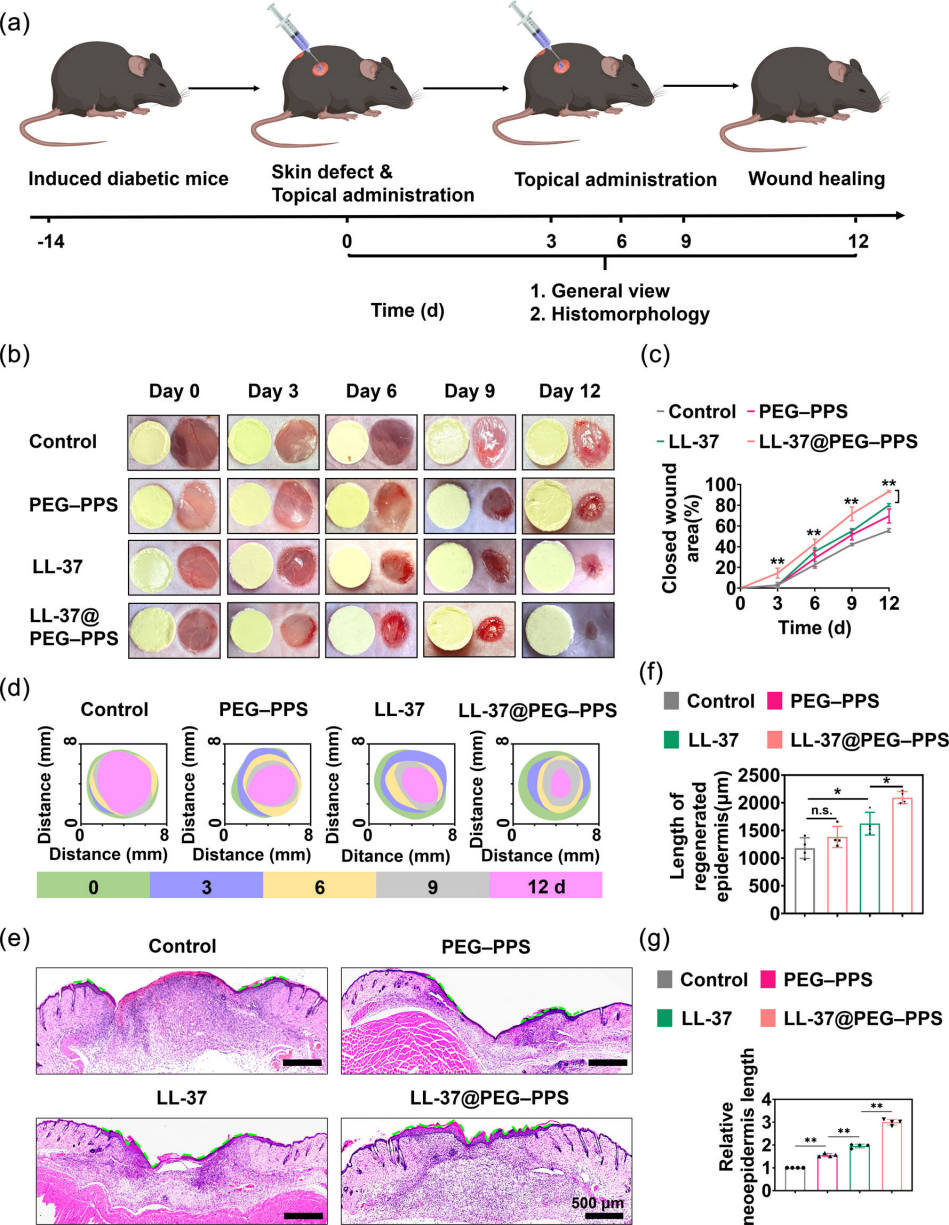
Therapeutic effects of LL‐37@PEG–PPS for diabetic wound healing. (a) Schematic diagram of the establishment and treatment of diabetic wounds. (b) Representative images of wound healing in diabetic mice on days 0, 3, 6, 9, and 12 after injury with different treatments. (c) Statistical analysis results of wound closure rate on days 0, 3, 6, 9, and 12 after injury with different treatments (*n* = 4). (d) Trace of wound closure following different treatments on days 0, 3, 6, 9, and 12 after the surgery. (e) On day 12 after injury, the length of the new epidermis in the different treatment groups is indicated by the green dashed line. (f) Data analysis of the neoepidermis length in the different treatment groups at day 12 (*n* = 4). (g) The ratio of the neoepidermis length in the different treatment groups to the neoepidermis length in the control group at day 12 (*n* = 4). The data in (c), (f), and (g) are expressed as mean ± SD. *p < 0.05, **p < 0.01, n.s., no significance (one‐way ANOVA). PEG–PPS, poly(ethylene glycol)–poly(propylene sulfide).

Type I diabetes was induced in mice to observe the therapeutic effect of LL‐37@PEG–PPS on diabetic chronic wounds. A round wound with a diameter of 6 mm was punched on the back of the mice. Then, 100 μL 2 mg LL‐37@PEG–PPS (containing 2.6 μg LL‐37 and 197 μg PEG–PPS) was topically applied to the wound surface on days 0, 3, and 6 after the surgery (Figure 5a). For comparison, 100 μL PBS, 200 μg PEG–PPS, and 2.6 μg LL‐37 were applied to the wound. The PBS‐treated group was regarded as the control group. A transparent Tegaderm film was fixed onto the wound surface. The wounds were photographed on days 0, 3, 6, 9, and 12 following the surgery to collect wound healing information and measure the wound size (Figure 5b). On day 12 following LL‐37@PEG–PPS treatment, the wound closure rate reached up to 93%, which exceeded that of the LL‐37 (79%, *p* < 0.01) and PEG–PPS (68%, *p* < 0.01) groups (Figure 5c,d).

The length of new epidermal tissue is an inherent index to determine the rate of wound healing (Figure 5e). On day 12, the epithelium was confluent in the LL‐37@PEG–PPS treatment group. The length of the new epidermis of the mice treated with LL‐37@PEG–PPS was greater than that of the mice treated with LL‐37 (Figure 5f,g) (*p* < 0.01). In order to evaluate the combined potential of LL‐37 and PEG–PPS, the BLISS model was used to analyze and confirm the synergistic effect of LL‐37 and PEG–PPS in promoting wound healing^37^ (Report S1).

**FIGURE 6:**
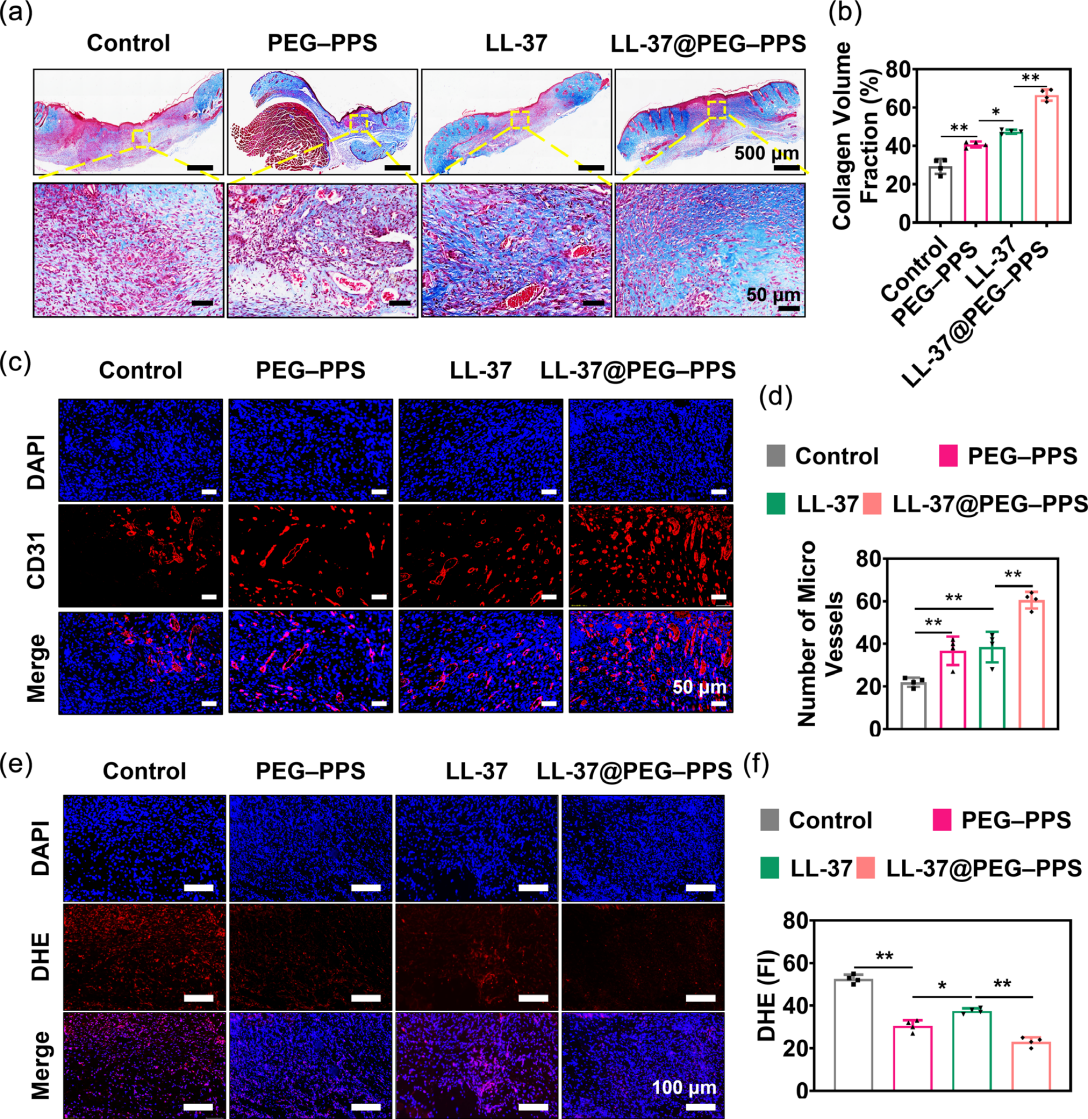
Therapeutic effects of LL‐37@PEG–PPS for diabetic wound healing. (a) Representative images of Masson staining for diabetic wounds under different treatments. (b) Statistical analysis of the total collagen volume fraction on day 12 following injury in the different treatment groups (*n* = 4). (c) CD31 and DAPI staining results of diabetic wound tissue in the different groups (scale bar: 50 μm). (d) Statistical analysis of the number of blood vessels in the wound tissue on day 12 (*n* = 4). (e) Confocal representative images of DHE (red) staining in the different treatment groups on day 9 (scale bar: 100 μm). (f) Fluorescence intensities of DHE (red) in the different treatment groups were analyzed using Image J software on day 9 (*n* = 4). The data in (b), (d), and (f) are expressed as mean ± SD. *p < 0.05, **p < 0.01 (one‐way ANOVA). DHE, dihydroethidium; PEG–PPS, poly(ethylene glycol)–poly(propylene sulfide).

The thickness of the new granulation tissue plays an important role in wound healing. The thickness of the granulation tissue in the control, PEG–PPS treatment, LL‐37 treatment, and LL‐37@PEG–PPS treatment groups were 410 ± 40, 702 ± 56, 788 ± 33, and 1013 ± 133 μm, respectively (Figure S16A,B). Collagen is an important index that reflects elasticity and toughness in the skin connective tissue.^17^, ^38^ Therefore, collagen deposition was also assessed via histological analysis (Figure 6a). On day 12, high collagen volume fraction was observed in the dermis in the LL‐37@PEG–PPS treatment group (Figure 6b). These results indicate that LL‐37@PEG–PPS treatment is beneficial for wound healing.

Blood vessels facilitate wound healing by delivering nutrients and oxygen to the wound tissue.^39^ Impaired wound healing in patients with diabetes is attributed to insufficient angiogenesis.^40^ As a transmembrane protein, CD31 can be specifically stained in newly formed blood vessels.^41^ To assess the effect of LL‐37@PEG–PPS in promoting angiogenesis at the diabetic wound site, immunofluorescence and immunohistochemical staining of CD31 was performed for the histological analysis of angiogenesis (Figure 6c and Figure S17). The number of neovascularizations in the LL‐37@PEG–PPS treatment group (60 ± 3) was significantly higher than that in the control (22 ± 2, *p* < 0.01), LL‐37 (39 ± 7, *p* < 0.01), and PEG–PPS groups (36 ± 3, *p* < 0.01) (Figure 6d). These results demonstrate that LL‐37@PEG–PPS exhibits better proangiogenesis effects than LL‐37. Blank PEG–PPS micelles can promote angiogenesis, possibly because of the alleviation of oxidative stress in the wound tissues, which indirectly regulates angiogenesis.

A chronic wound is characterized by excessive ROS accumulation, creating a hostile microenvironment for wound healing.^42^ A DHE probe was used to evaluate the ROS level of the wound tissue.^43^ On the day 9 after injury, ROS accumulation at the wound tissue of the control group considerably increased. However, the DHE signal (red fluorescence) was weaker than that in the LL‐37 treatment group (*p* < 0.01) and control group (*p* < 0.01) (Figure 6e,f), demonstrating that ROS decreased following LL‐37@PEG–PPS treatment.

**FIGURE 7:**
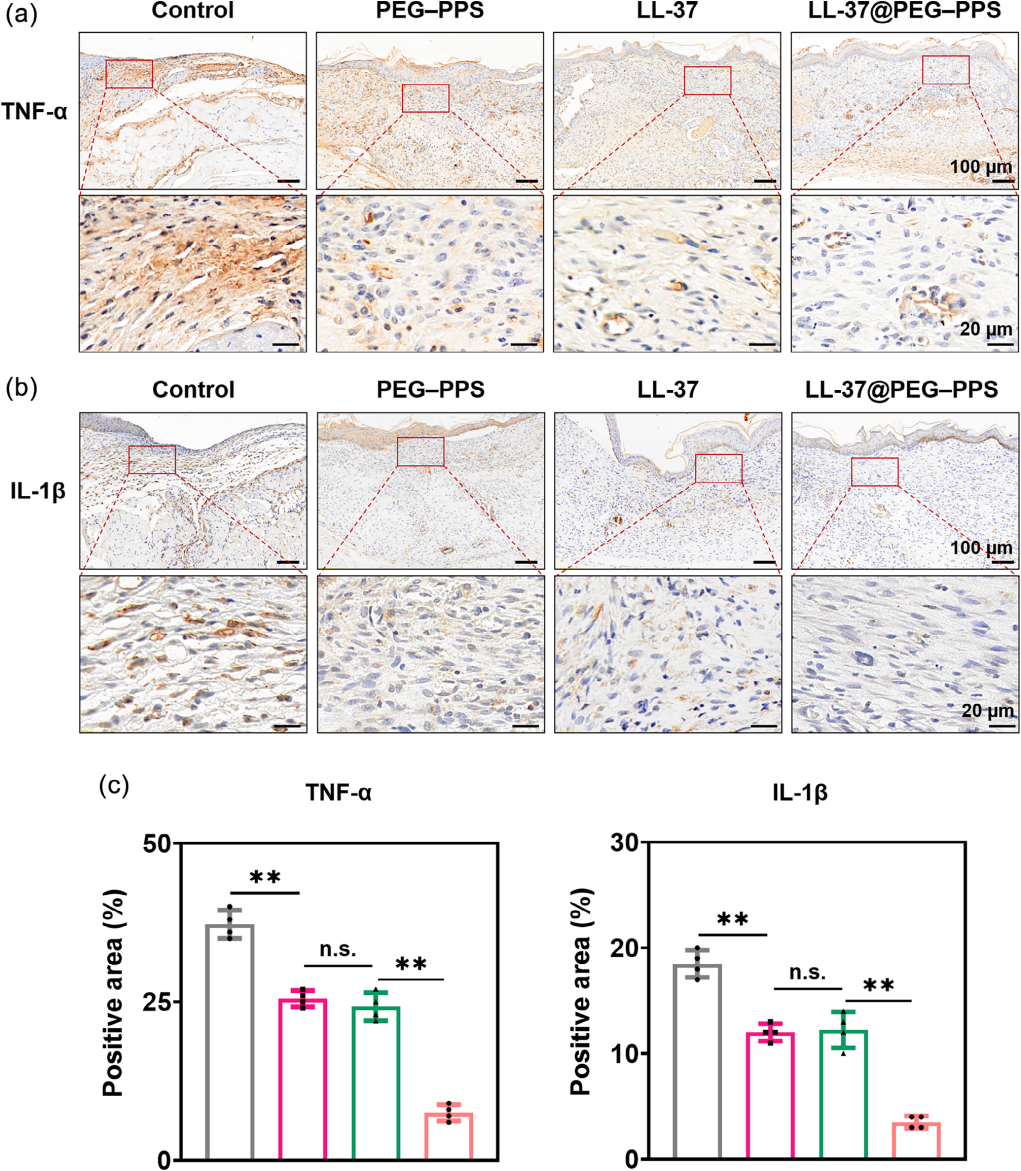
Immunohistochemical staining of the inflammatory factors TNF‐α and IL‐1β in diabetic wounds. (a) Immunohistochemical staining of TNF‐α in diabetic wounds under different treatments. (b) Immunohistochemical staining of IL‐1β of diabetic wounds under different treatments. (c) Statistical analysis of the percentage of TNF‐α‐ and IL‐1β‐positive area in wound tissue (*n* = 4). The data are expressed as mean ± SD. *p < 0.05, **p < 0.01, n.s., no significance (one‐way ANOVA). IL, interleukin; TNF, tumor necrosis factor.

Chronic diabetic wounds exhibit imbalance and persistent M1 (proinflammatory) macrophage polarization. However, the macrophages in a normal wound transition to the M2 phenotype on the third day following injury.^44^ The proinflammatory macrophages (M1) continuously release proinflammatory cytokines, such as TNF‐α and IL‐1β, thereby exacerbating tissue damage and inflammation.^45^ The transition of macrophages from the M1 to M2 phenotype is crucial for wound repair and tissue regeneration. LL‐37@PEG–PPS decreased the proinflammatory M1 phenotype (downregulated TNF‐α and IL‐1β; Figure 7a,b), whereas the expression level of TNF‐α and IL‐1β in the control group was significantly higher than that in other treatment groups (Figure 7c), indicating that LL‐37@PEG–PPS can downregulate proinflammatory cytokines such as TNF‐α and IL‐1β and effectively alleviate wound inflammation.

These results indicate that LL‐37@PEG–PPS scavenges ROS and alleviates inflammation in the wound, further reducing ROS generation in the wound and producing a good microenvironment for chronic wound healing. In addition, LL‐37@PEG–PPS not only improved the wound microenvironment but also protected LL‐37 from degradation, thereby providing LL‐37 peptides the opportunity to exert their biological functions and jointly promoting wound healing.

### 3.7 Biocompatibility of LL‐37@PEG–PPS

**FIGURE 8:**
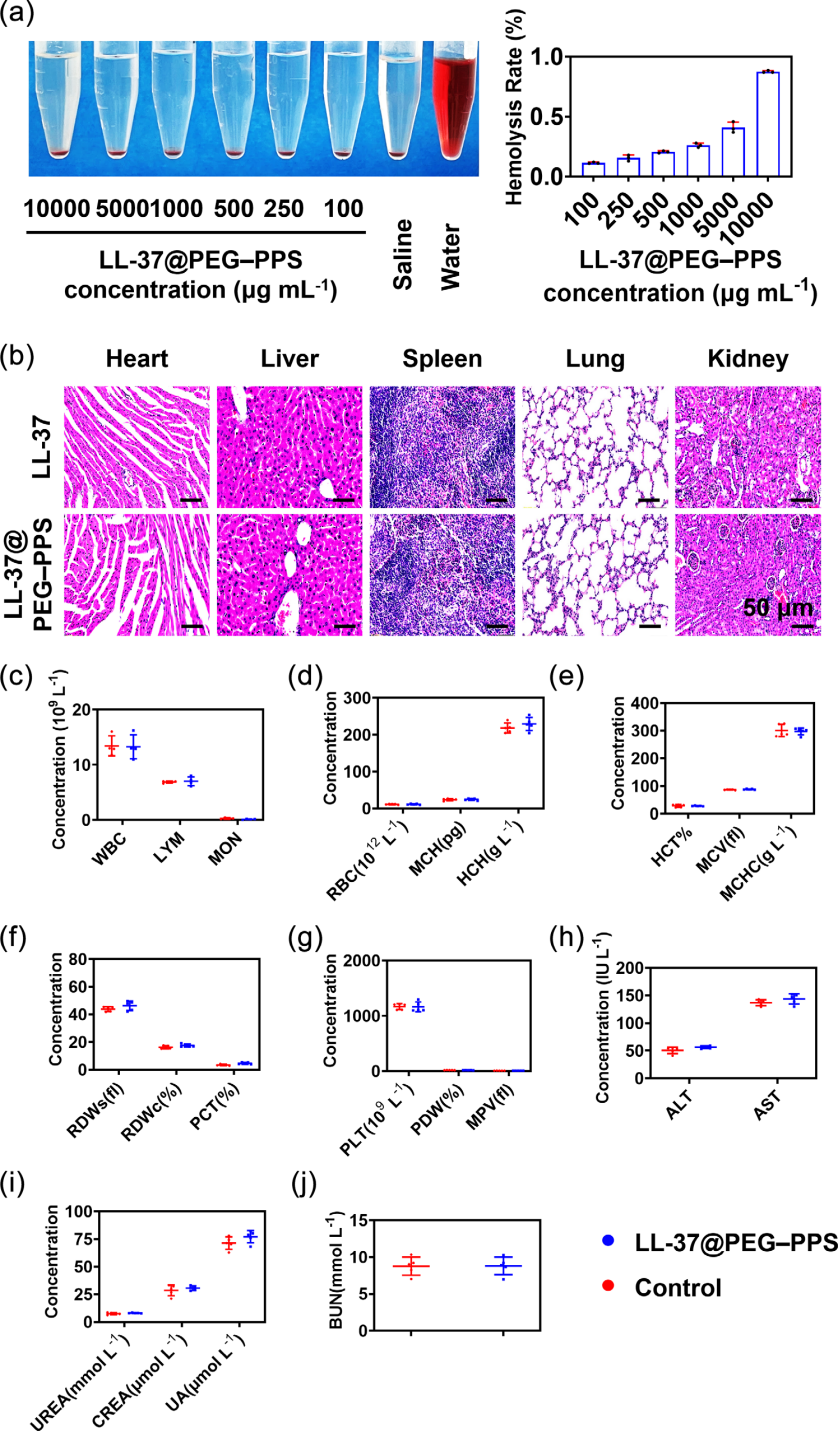
Biocompatibility of LL‐37@PEG–PPS nanomicelles in vitro and in vivo. (a) Typical photographs and hemolysis rate of red blood cells incubated with LL‐37@PEG–PPS in vitro at different concentrations (*n* = 3). (b) On day 12 after surgery, the toxicity evaluation of LL‐37@PEG–PPS in the major organs (heart, liver, spleen, lungs, and kidneys) was performed. Scale bar: 50 μm. (c–g) Routine blood analysis of mice treated with PBS and LL‐37@PEG–PPS. (h) Analysis of liver function in mice treated with PBS and LL‐37@PEG–PPS. (i and j) Renal function analysis of mice treated with PBS and LL‐37@PEG–PPS (*n* = 5). PEG–PPS, poly(ethylene glycol)–poly(propylene sulfide).

The cytocompatibility of LL‐37@PEG–PPS in HUVECs was evaluated via the CCK‐8 assay. When the concentration of LL‐37@PEG–PPS was 15–500 μg mL^−1^, it was safe for HUVECs and exerted no cytotoxicity (Figure S18). When the concentration of LL‐37@PEG–PPS was 1000 μg mL^−1^, the cell viability remained up to 77%. Subsequently, the blood compatibility of LL‐37@PEG–PPS was evaluated using a hemolysis test. According to the international evaluation standard, the hemolysis rate of biological materials for clinical use should be <5%.^46^ At a concentration of 1000 μg mL^−1^, the hemolysis rate of LL‐37@PEG–PPS was only 0.2% (Figure 8a).

To further examine the toxicity of LL‐37@PEP–PPS in vivo, 200 mg kg^−1^ (6.7 times the concentration of LL‐37@PEG–PPS used to treat diabetic wounds) was topically applied to the wound surface of diabetic mice on days 0, 3, and 6, respectively. The control group was treated with normal saline. The whole blood was harvested for serum examination, and the animals were sacrificed on day 12. The main organs (heart, liver, spleen, lung and kidney) were collected for histopathology. No bleeding, ischemia, necrosis, or infarction was observed in the heart, liver, spleen, lung, and kidney tissues (Figure 8b). No abnormalities were observed in the routine blood and biochemical test results. The related indices of liver and renal functions in the LL‐37@PEG‐PPS treatment group were close to those in the control group (*p* > 0.01) (Figure 8c–j). These results provide evidence that LL‐37@PEG–PPS possesses good biocompatibility.

Chronic diabetic wounds present a huge economic burden on individuals and society. Although various treatments have considerably improved the healing of diabetic wounds in the past decades, their treatment continues to face enormous challenges and new interventions are urgently required.^47^ After almost two decades (Regranex was approved in 1997),^48^ no new chemical entities have been approved by the Food and Drug Administration; thus, it has become apparent that an optimal wound‐healing outcome will require a multifaceted approach that can simultaneously address various issues such as persistent inflammation, insufficient angiogenesis, and impaired reepithelialization. Although some bioactive molecules such as growth factors and stem cells have been used in the clinical treatment of diabetic wounds, they are associated with problems such as poor stability, easy inactivation, low bioavailability, and single regulation of wound microenvironment.

We have developed an LL‐37‐loaded nanocarrier based on the assembly of PEG–PPS diblock copolymers and the LL‐37 polypeptide. For chronic wound healing, more ROS scavengers may be needed to balance the level of ROS on the wound surface. In vitro experiments revealed that LL‐37@PEG–PPS effectively scavenged intracellular ROS and protected cells against apoptosis.

Angiogenesis plays a crucial role in diabetic wound healing. Neovascularization provides oxygen and nutrition to the wound, thereby accelerating wound healing.^49^ Several studies have demonstrated that LL‐37 can promote vascular endothelial cell migration and angiogenesis. Through tube‐formation and scratch experiments, LL‐37@PEG–PPS was observed to effectively promote angiogenesis. As VEGF plays an important role in angiogenesis, we evaluated the effects of different treatments on VEGF secretion ability of HUVECs. The high level of VEGF secreted by the LL‐37@PEG–PPS group confirms its advantage in promoting angiogenesis.

Currently, several nanoparticles related to LL‐37 have demonstrated certain effects in angiogenesis (Table 2). Comune et al. reported a soluble and immobilized LL‐37 (LL‐37‐conjugated gold nanoparticles) in a splinted mouse full‐thickness excisional model, which can increase the number of new blood vessels by 2.30 times.^50^ Yang et al. reported that chitosan hydrogel encapsulated with LL‐37 peptide increased the number of new blood vessels 2.21 times in a deep tissue damage mouse model.^32^ Wang et al. reported that antimicrobial peptide (LL‐37) grafted with ultrasmall gold nanoparticles increased the number of new blood vessels 1.83 times in the wound model of diabetic mouse.^13^ Herein, we developed a dual functional therapeutic agent based on the assembly of LL‐37 peptides and PEG–PPS, which increased the number of new blood vessels by 2.75 times of control group in a wound model of diabetic mouse. With an LL‐37 LC of 1.3%, LL‐37@PEG–PPS can promote angiogenesis in the wound tissue 2.75 times of that in the control group, which is superior to the angiogenesis effects reported in the studies cited in Table 2. This highlights the protective effect of PEG–PPS on LL‐37. The nanomicelle platform transforms an unfavorable wound microenvironment into a favorable one by scavenging excessive ROS; and the controlled release of LL‐37 induces the sprouting of new blood vessels, thereby improving angiogenesis and accelerating diabetic wound healing.

**TABLE 2:** The angiogenesis effects of LL‐37 in Refs.

No.	Ref.	Carrier of LL‐37	Day	Microvessels number to control
1	*J. Control. Release*. 2017;262:58–71	AuNPs	10	2.30 fold
2	*Mil. Med. Res*. 2020;7(1):20	Chitosan hydrogel	14	2.21 fold
3	*Biomater. Sci*. 2018;6(10):2757–2772	AuNPs/pDNAs	10	1.83 fold
4	This work	PEG–PPS	12	2.75 fold

Abbreviation: PEG–PPS, poly(ethylene glycol)–poly(propylene sulfide).

Our proposed treatment not only scavenged excessive ROS, which improves the microenvironment for angiogenesis, but also released LL‐37 peptides on demand and protected them from degradation, resulting in a robust increase in angiogenesis, the restoration of beneficial angiogenesis in the wound microenvironment, continuous provision of signals to promote angiogenesis and epidermal cell migration, and accelerated and high‐quality wound healing in vivo. Thus, the in vivo and in vitro results successfully confirmed that LL‐37@PEG–PPS exhibits good biocompatibility, suggesting its future clinical application prospects. Although LL‐37@PEG–PPS demonstrates good effects in chronic wound healing, the problem of the easy degradability of LL‐37 remains unresolved. In addition, the LC of LL‐37 is not particularly efficient. In the future, we should further optimize the structure of the PEG‐PPS, improve the LC of LL‐37, and enhance the combination mode of the material and LL‐37 to better protect the biological activity of LL‐37 and maximize its biological role. We expect that our findings will provide a promising solution for treating chronic refractory wounds.

## 4 CONCLUSION

We developed LL‐37@PEG–PPS nanomicelles via the self‐assembly of LL‐37 and PEG–PPS, which improved the microenvironment and promoted diabetic wound healing. PEG–PPS can effectively load the LL‐37 polypeptide and protect it from degradation to avoid biological function failure. The nanomicelles exhibited a good ability to transform the oxidatively damaged microenvironment into a regenerative microenvironment and provide signals for angiogenesis. They could react to excessive ROS accumulation in cells and release LL‐37 on demand, thus contributing to angiogenesis and accelerating wound healing.

## AUTHOR CONTRIBUTIONS

**Rong Shi:** Conceptualization (equal); data curation (equal); formal analysis (equal); investigation (equal); methodology (equal); resources (equal); validation (equal); visualization (equal); writing – original draft (equal); writing – review and editing (equal). **Jianxiong Qiao:** Conceptualization (equal); data curation (equal); formal analysis (equal); investigation (equal); methodology (equal); resources (equal); validation (equal); visualization (equal); writing – original draft (equal); writing – review and editing (equal). **Quanwu Sun:** Investigation (equal); validation (equal); writing – review and editing (equal). **Biao Hou:** Investigation (equal); validation (equal); writing – review and editing (equal). **Bo Li:** Investigation (equal); validation (equal); writing – review and editing (equal). **Ji Zheng:** Investigation (equal); writing – review and editing (equal). **Zhenzhen Zhang:** Investigation (equal); writing – review and editing (equal). **Zhenxue Peng:** Investigation (equal); writing – review and editing (supporting). **Jing Zhou:** Investigation (equal); writing – review and editing (supporting). **Bingbing Shen:** Conceptualization (equal); funding acquisition (equal); project administration (equal); supervision (equal); writing – original draft (equal); writing – review and editing (equal). **Jun Deng:** Conceptualization (equal); funding acquisition (equal); project administration (equal); supervision (equal); writing – original draft (equal); writing – review and editing (equal). **Xuanfen Zhang:** Conceptualization (equal); funding acquisition (equal); project administration (equal); supervision (equal); writing – original draft (equal); writing – review and editing (equal).

## CONFLICT OF INTEREST STATEMENT

The authors declare no conflicts of interest.

## Supporting information


**Data S1.** Supporting Information.

## Data Availability

All data that supports this review are available upon reasonable request from the co‐corresponding authors.
