# Correction to “Self‐assembly of PEG–PPS polymers and LL‐37 peptide nanomicelles improves the oxidative microenvironment and promotes angiogenesis to facilitate chronic wound healing”

**DOI:** 10.1002/btm2.10718

**Published:** 2024-09-09

Shi R, Qiao J, Sun Q, Hou B, Li B, Zheng J, Zhang Z, Peng Z, Zhou J, Shen B, Deng J, Zhang X. Self‐assembly of PEG–PPS polymers and LL‐37 peptide nanomicelles improves the oxidative microenvironment and promotes angiogenesis to facilitate chronic wound healing. *Bioeng Transl Med*. 2023;9(2):e10619. doi:10.1002/btm2.10619

The authors regret some errors have been found in Figure 5, Figure S12, and Figure S15.

In Figure 5, due to the misuse of wound images of the LL‐37@PEG–PPS group on day 9, there was a duplication with the wound images of the PEG–PPS group on day 11.

In Figure S12a, due to misuse of images, there was partial overlap of the 0 h images between the control group and PEG–PPS group.

In Figure S15a, unintentional misuse of the in vivo biodistribution image of FITC‐LL‐37@PEG–PPS in before injection group, which leads to an overlapped with that on day 4.

The corrected image and its original image captions are shown below. This error will not affect the main conclusion. The authors sincerely apologize for any inconvenience and confusion caused.

**Figure:**
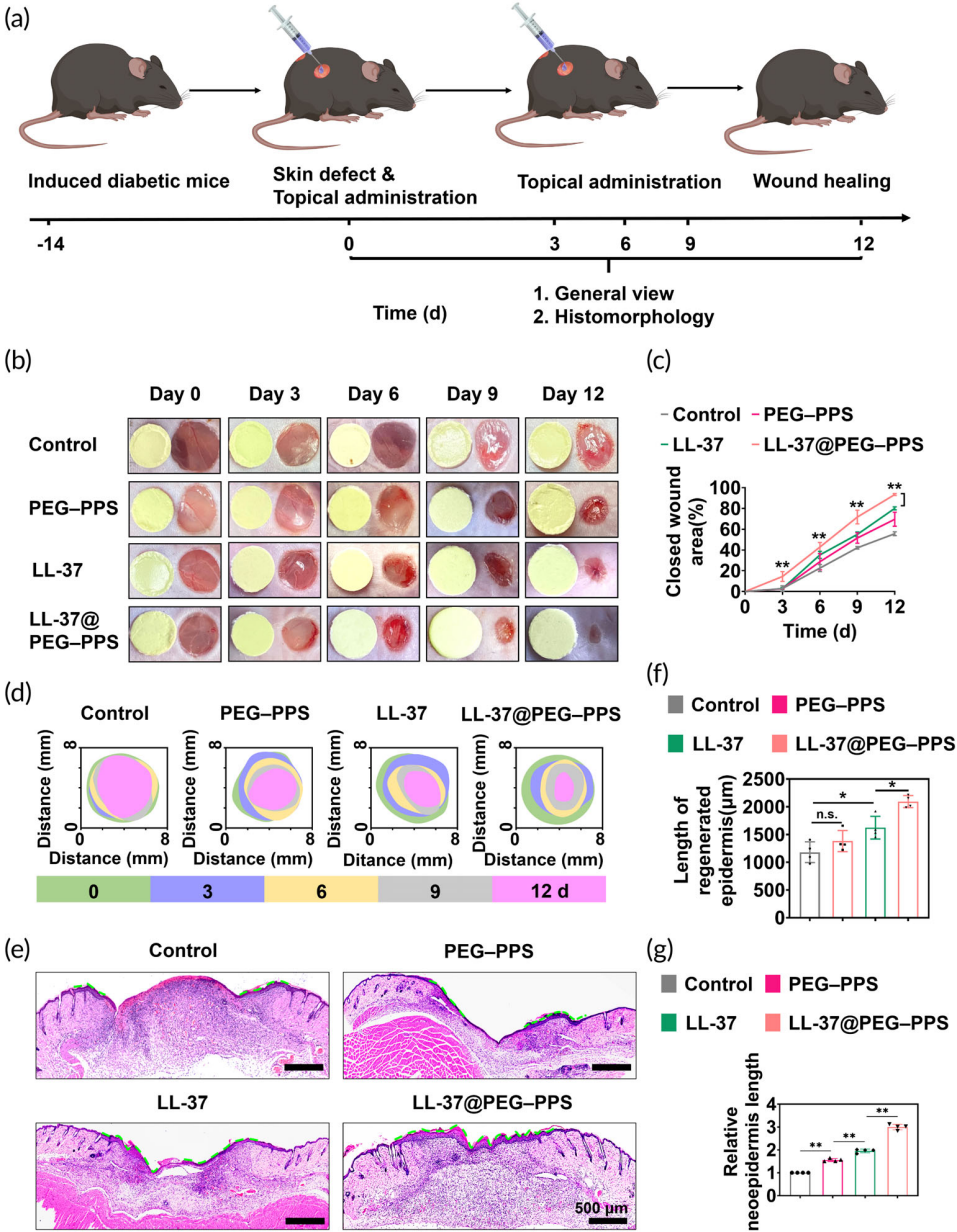
**FIGURE 5** Therapeutic effects of LL‐37@PEG–PPS for diabetic wound healing. (a) Schematic diagram of the establishment and treatment of diabetic wounds. (b) Representative images of wound healing in diabetic mice on days 0, 3, 6, 9, and 12 after injury with different treatments. (c) Statistical analysis results of wound closure rate on days 0, 3, 6, 9, and 12 after injury with different treatments (*n* = 4). (d) Trace of wound closure following different treatments on day 0, 3, 6, 9, and 12 after the surgery. (d) On day 12 after injury, the length of the new epidermis in the different treatment groups is indicated by the green dashed line. (f) Data analysis of the neoepidermis length in the different treatment groups at day 12 (*n* = 4). (g) The ratio of the neoepidermis length in the different treatment groups to the neoepidermis length in the control group at day 12 (*n* = 4). The data in c, f, and g are expressed as mean ± s.d. *p < 0.05, **p < 0.01, n.s., no significance, (one‐way ANOVA).

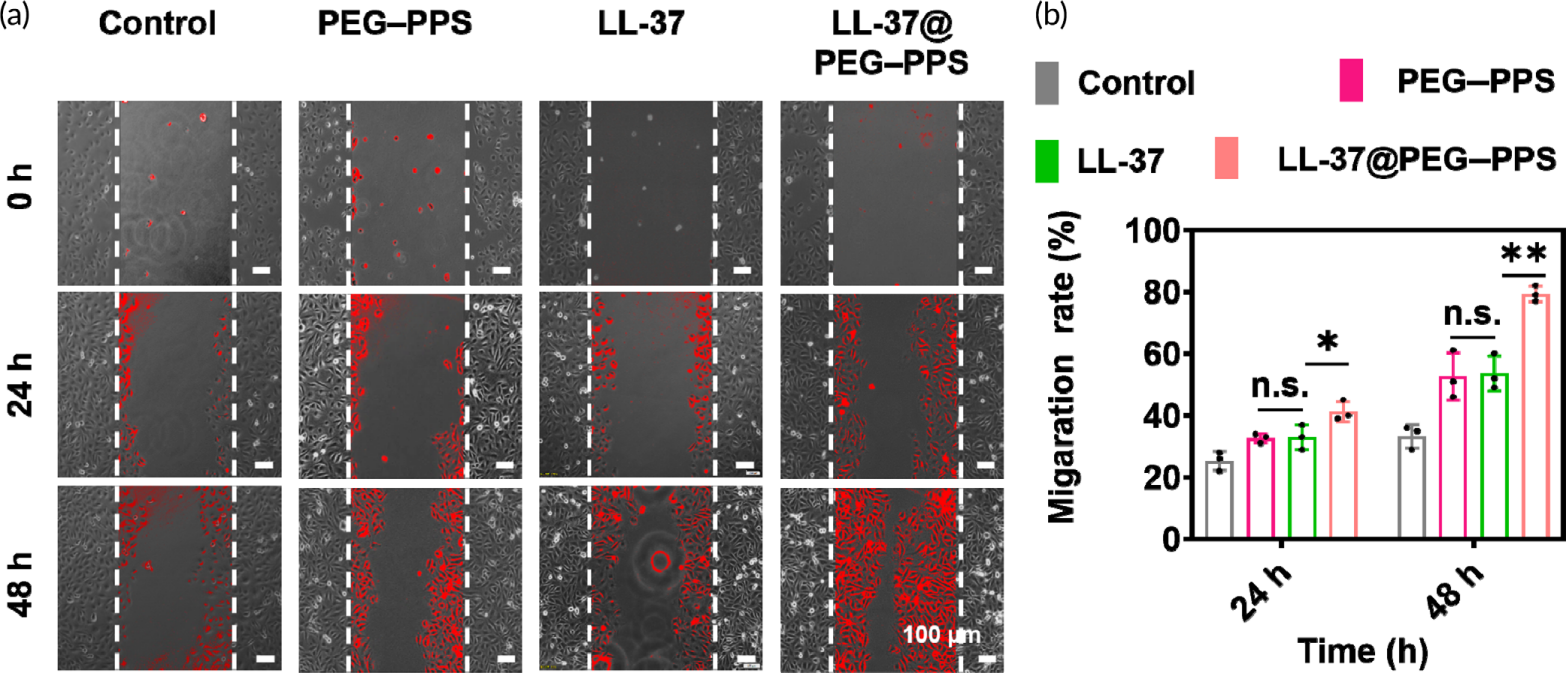

**FIGURE S12** (a) Representative images of scratch tests for HaCaT cells under PBS, LL‐37, PEG–PPS, and LL‐37@PEG–PPS treatments and quantitative analysis of migration (b). Data are represented as mean ± SD (*n* = 3). *p < 0.05, **p < 0.01, n.s., no significance, (one‐way ANOVA).

**Figure:**
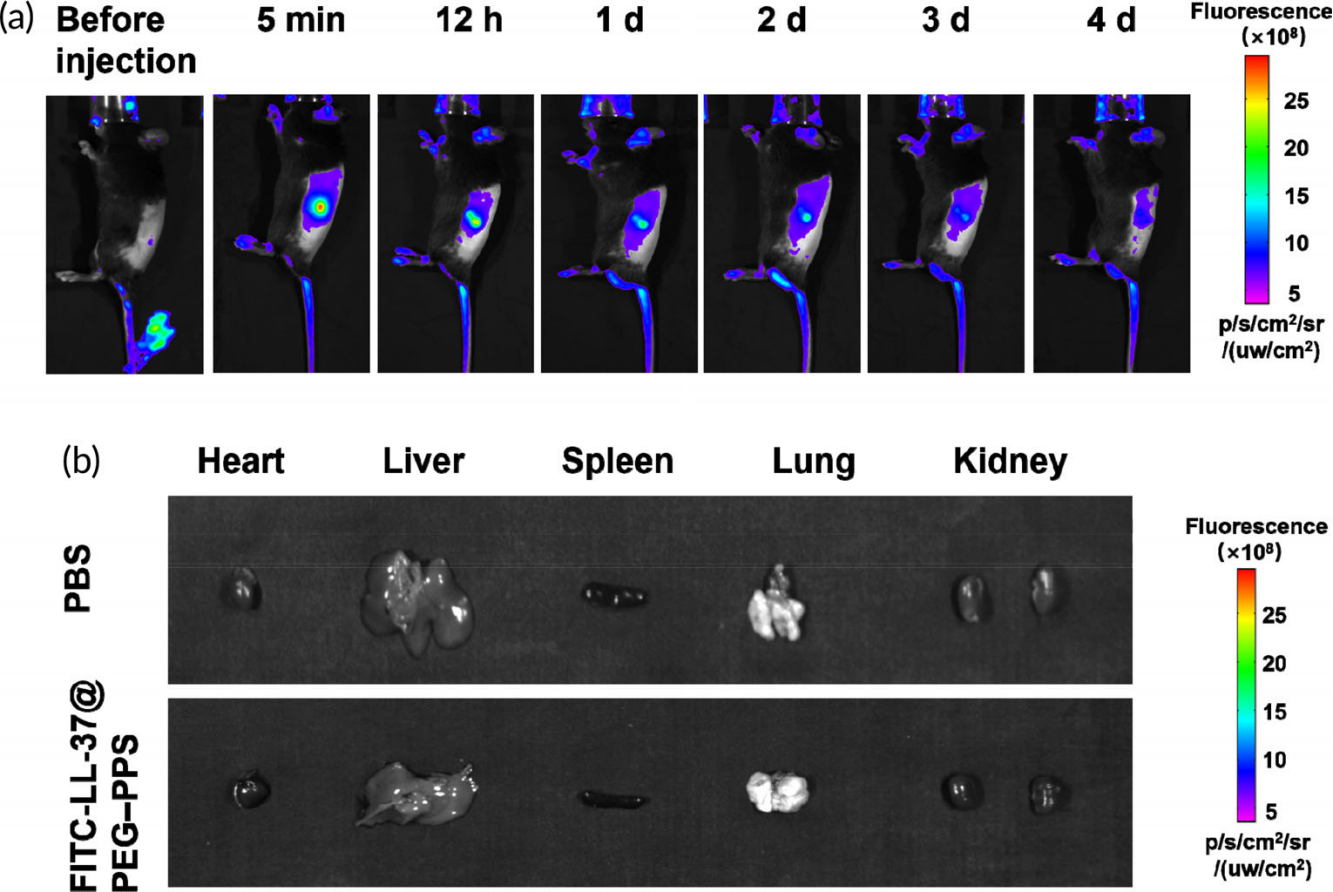
**FIGURE S15** The biodistribution of FITC‐LL‐37@PEG–PPS in vivo. (a) The distribution of FITC‐LL‐37@PEG–PPS in vivo at different time points after subcutaneous injection. (b) On day 4 subcutaneous injection of FITC‐LL‐37@PEG–PPS, the distribution image of FITC‐LL‐37@PEG–PPS in the main organs in vitro.

