# Supplementary material for: Self‐assembly of PEG–PPS polymers and LL‐37 peptide nanomicelles improves the oxidative microenvironment and promotes angiogenesis to facilitate chronic wound healing
Source: Bioeng Transl Med. 2023 Nov 8;9(2):e10619. doi: 10.1002/btm2.10619 (PMC10905545; doi:10.1002/btm2.10619)

***Supplement information***

**Self-assembly of PEG–PPS polymers and LL-37 peptide nanomicelles improves the oxidative microenvironment and promotes angiogenesis to**

**facilitate chronic wound healing**

Rong Shi^a,b,1^, Jianxiong Qiao^a,1^, Quanwu Sun^b^, Biao Hou^c^, Bo Li^c^, Ji Zheng^d^, Zhenzhen Zhang^a^, Zhenxue Peng^a^, Jing Zhou^a^, Bingbing Shen^e,^*, Jun Deng^f,^*, Xuanfen Zhang^a,^*.

^a^Department of Plastic Surgery, Lanzhou University Second Hospital. Lanzhou, Gansu 730000, China

^b^Department of Breast Surgery, Gansu Provincial Hospital. Lanzhou, Gansu 730030, China

^c^Department of Joint Surgery and Sports Medicine, Center for Orthopedic Surgery, Orthopedic Hospital of Guangdong Province, The Third Affiliated Hospital of Southern Medical University. Guangzhou, Guangdong 510515, China

^d^Department of Urology, Xinqiao Hospital, Third Military Medical University (Army Medical University), Chongqing ,400037, China.

^e^Department of Nephrology，Chongqing University Central Hospital, Chongqing Emergency Medical Center, NO.1 Jiankang Street, Yuzhong District, Chongqing, 400014, China

^f^Institute of Burn Research, State Key Lab of Trauma, Burn, and Combined Injury, Chongqing Key Laboratory for Disease Proteomics, Southwest Hospital, Third Military Medical University (Army Medical University), Chongqing, 400038, China

^*^ Corresponding authors.

Email:shenbingbing@cqu.edu.cn (B. Shen); djun.123@163.com (J. Deng); zhxf9304@126.com (X. Zhang).

^1^ These authors contributed equally to this work.


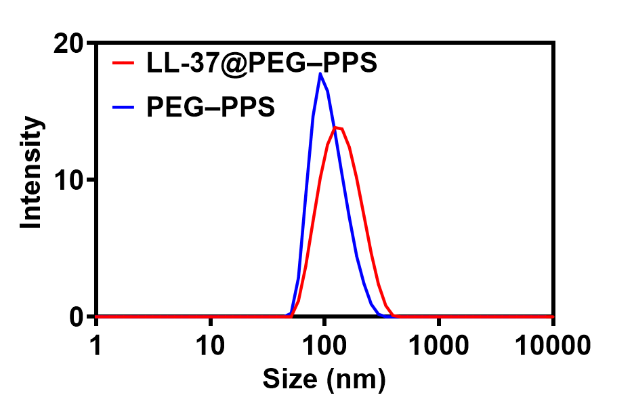


**Figure S1.** Hydrodynamic diameter of PEG–PPS and LL-37@PEG–PPS nano-micelles.


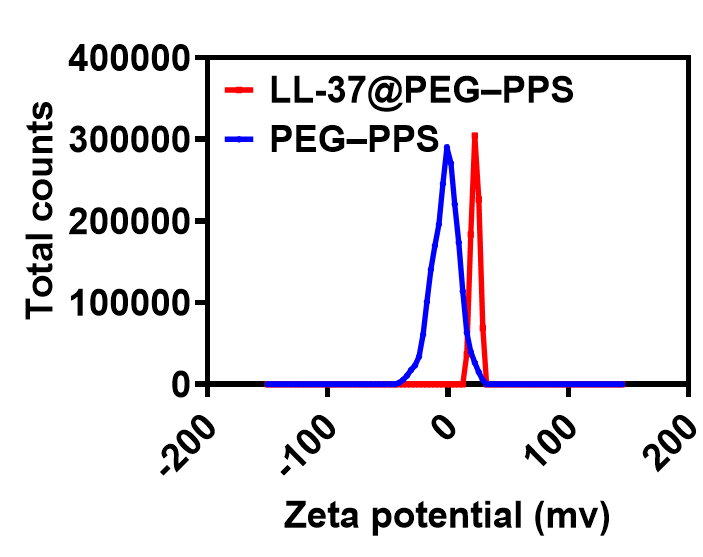


**Figure S2.** Zeta potential of PEG–PPS and LL-37@PEG–PPS.


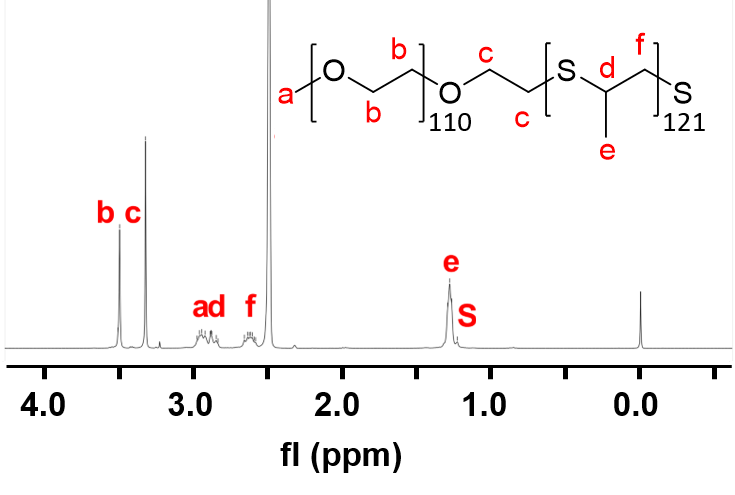


**Figure S3.** ^1^H NMR spectra of PEG–PPS in CDCl_3_.


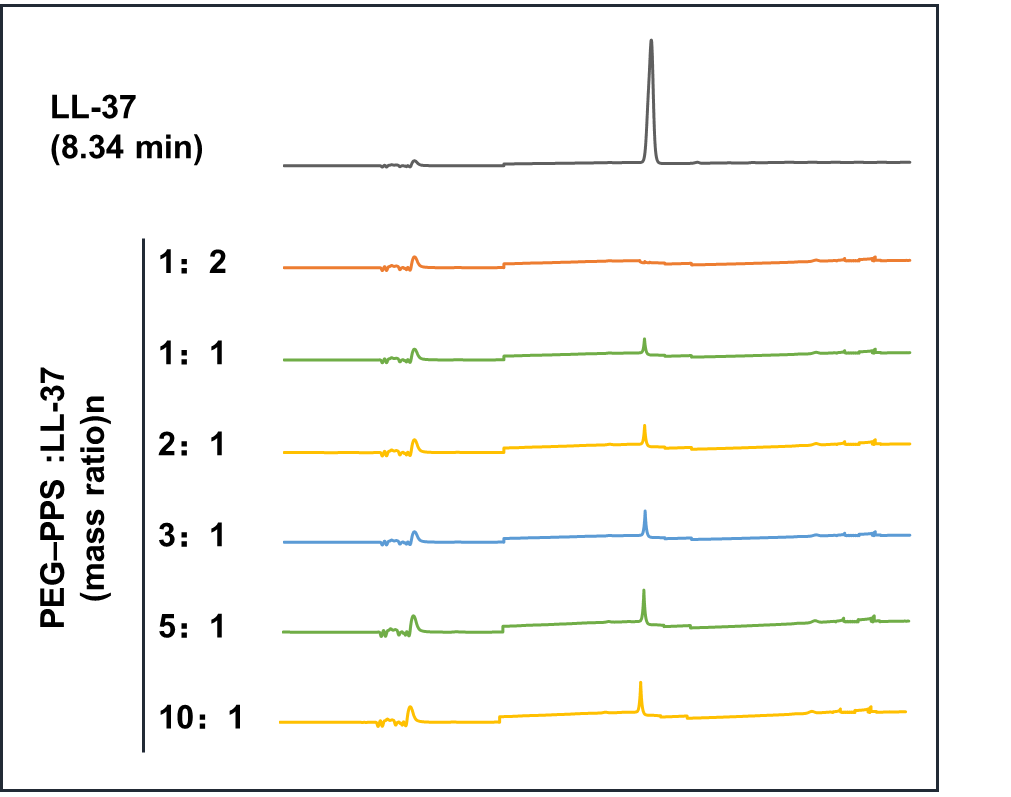


**Figure S4.** HPLC monitoring of LL-37 loading capacity of PEG–PPS to LL-37 at different mass ratios at a wavelength of 214 nm.


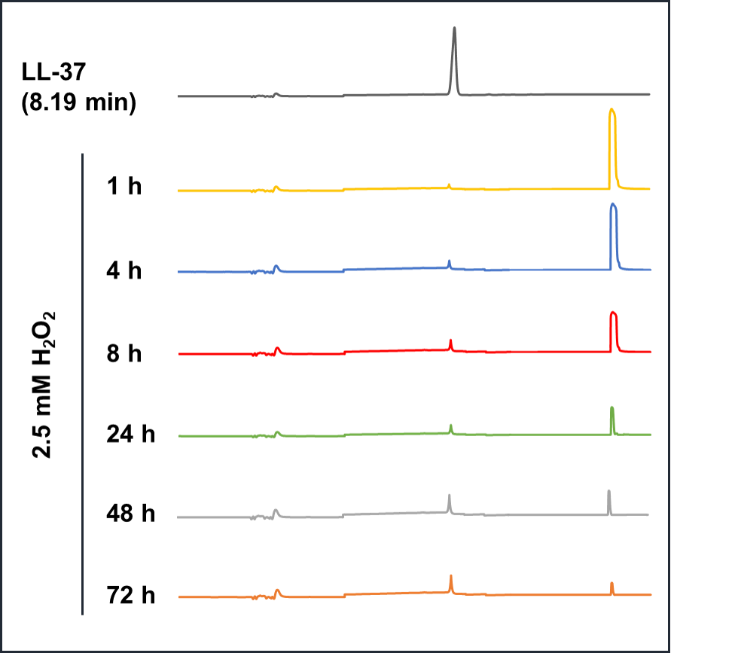


**Figure S5.** HPLC monitoring of the release of LL-37 at different time points at a wavelength of 214 nm.


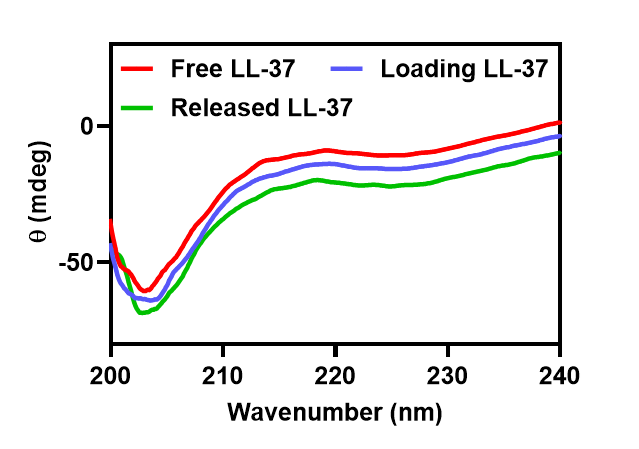


**Figure S6**. Circular dichroism (CD) analysis of free LL-37, loading LL-37, and released LL-37 peptides by LL-37@PEG–PPS.


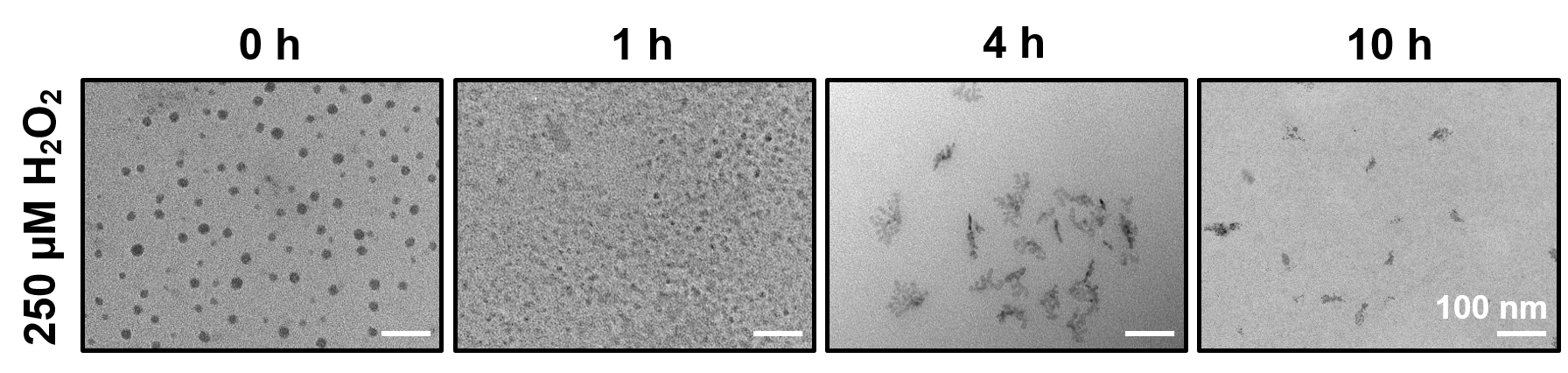


**Figure S7.** TEM images of LL-37@PEG–PPS nanomicelles after treatment with 250 µM H_2_O_2_ for 0, 1, 4, and 10 h. Scale bar: 100 nm.


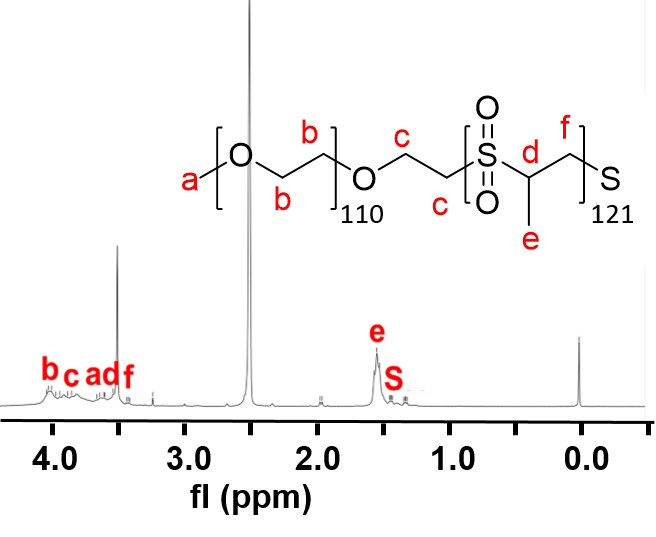


**Figure S8.** ^1^H NMR spectra of oxidative product of PEG–PPS in CDCl_3_.

_
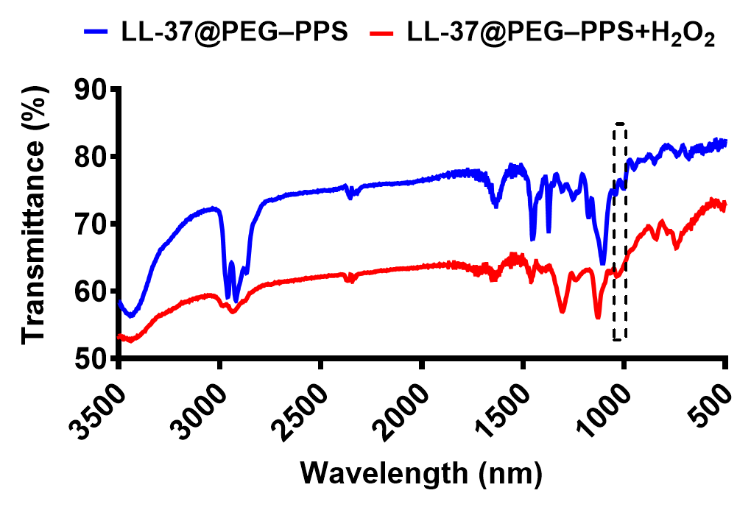
_

**Figure S9.** FTIR spectra of LL-37@PEG–PPS (blue) and the oxidative product of LL-37@PEG–PPS (red).


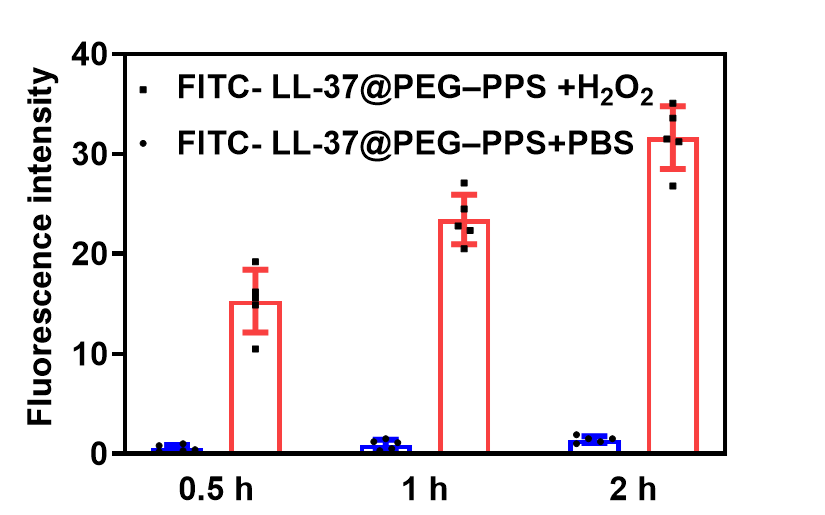


**Figure S10.** Statistical analysis of fluorescence intensity of FITC- LL-37@PEG–PPS in HUVEC cells was performed using ImageJ software. Data represent means ± s.d. n = 5.


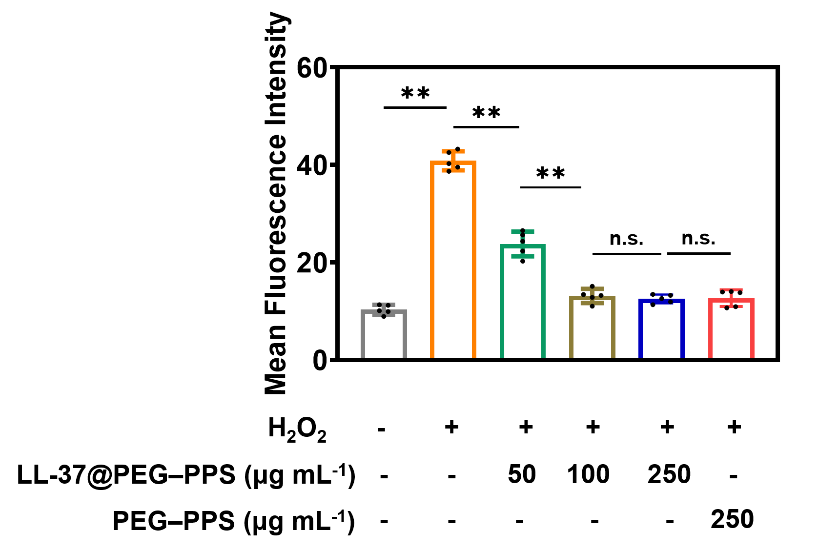


**Figure S11.** The mean fluorescence intensity of ROS (green) in different treatment groups was analyzed using Image J software. Data are represented as mean ± SD (n = 5). **p* < 0.05, ***p* < 0.01, n.s., no significance, (one-way ANOVA).


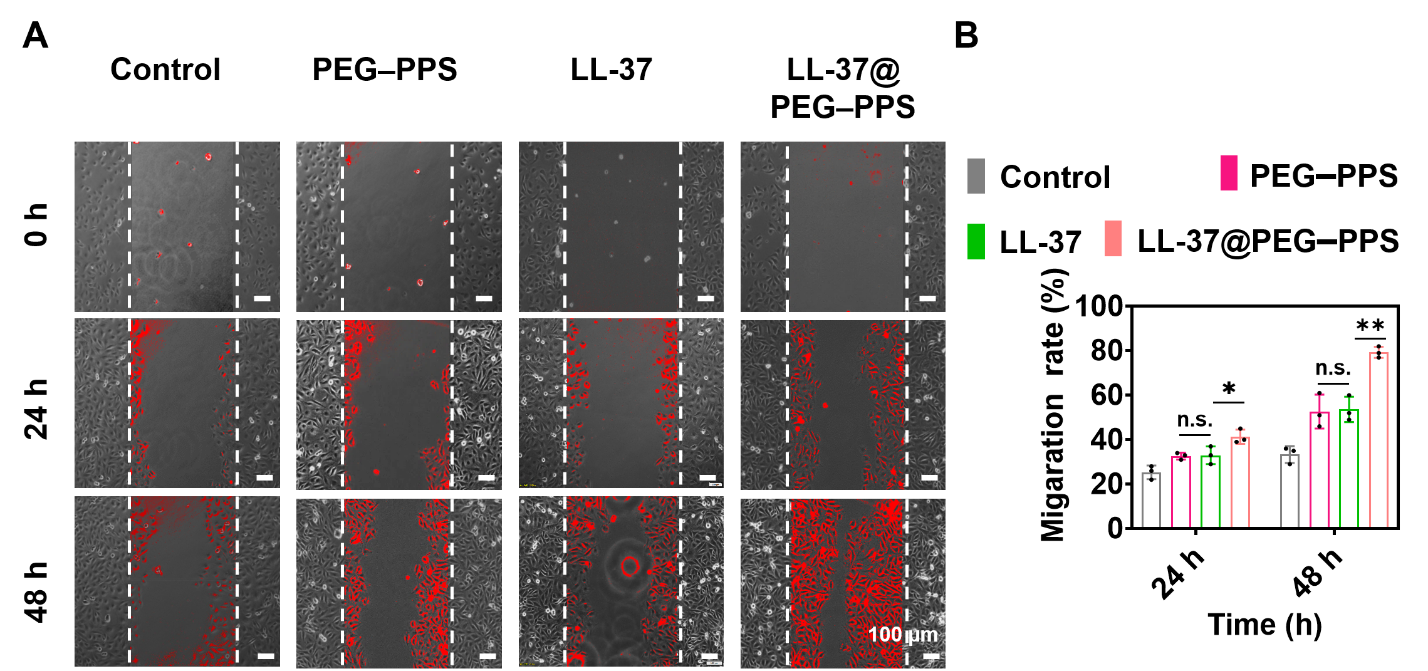


**Figure S12.** (A) Representative images of scratch tests for HaCaT cells under PBS, LL-37, PEG–PPS, and LL-37@PEG–PPS treatments and quantitative analysis of migration (B). Data are represented as mean ± SD (n = 3). **p* < 0.05, ***p* < 0.01, n.s., no significance, (one-way ANOVA).


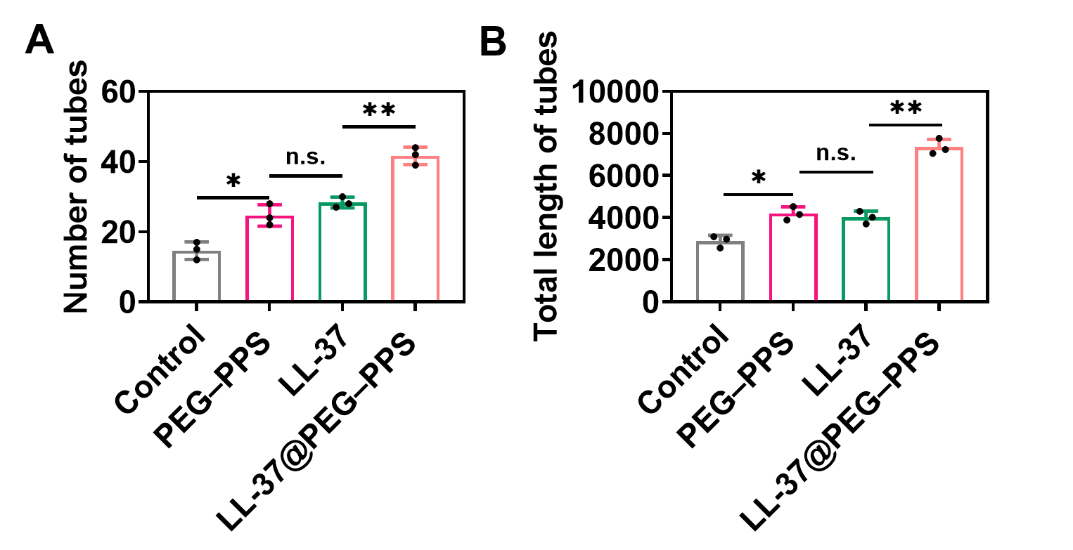


**Figure S13.** Quantitative analysis of the number of tubes and total length of tubes. Data are represented as mean ± SD (n = 3). **p* < 0.05, ***p* < 0.01, n.s., no significance, (one-way ANOVA).


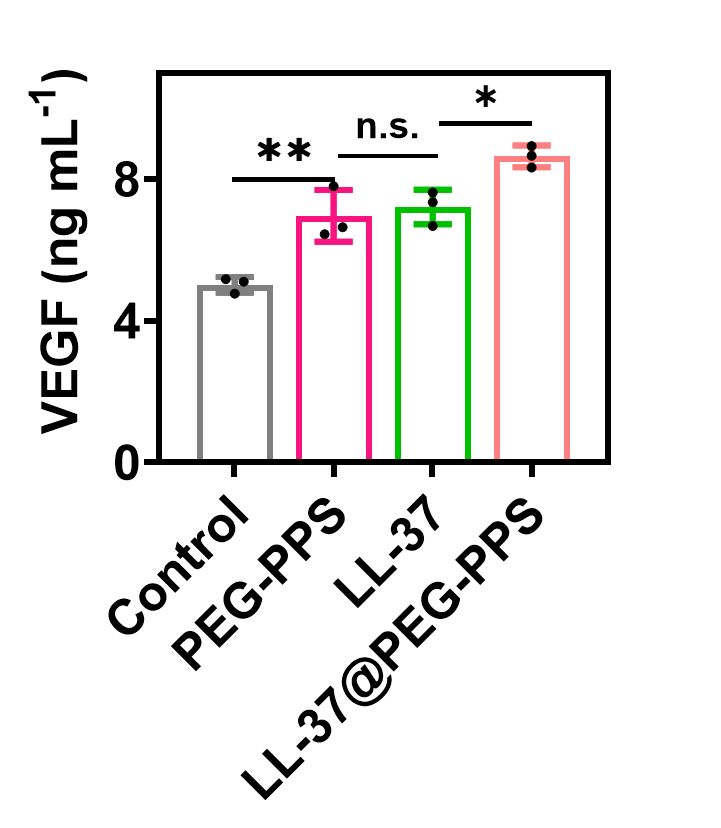


**Figure S14.** The content of VEGF in the supernatant of HUVECs treated with different treatments for 48 hours. Data are represented as mean ± SD (n = 3). **p* < 0.05, ***p* < 0.01, n.s., no significance, (one-way ANOVA).


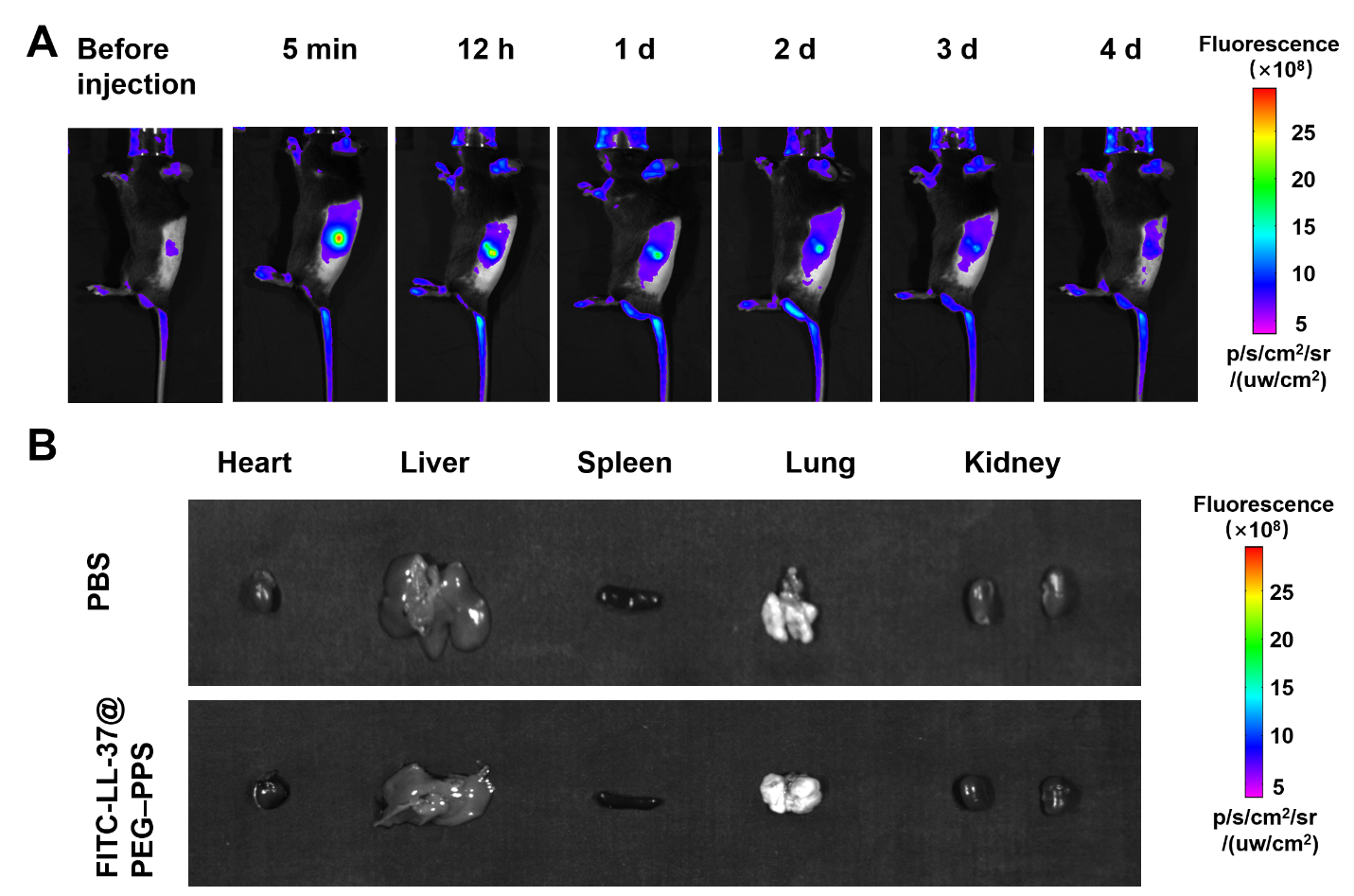


**Figure S15. The biodistribution of FITC-LL-37@PEG–PPS *in vivo*.** (A) The distribution of FITC-LL-37@PEG–PPS *in vivo* at different time points after subcutaneous injection. (B) On day 4 subcutaneous injection of FITC-LL-37@PEG–PPS, the distribution image of FITC-LL-37@PEG–PPS in the main organs *in vitro*.


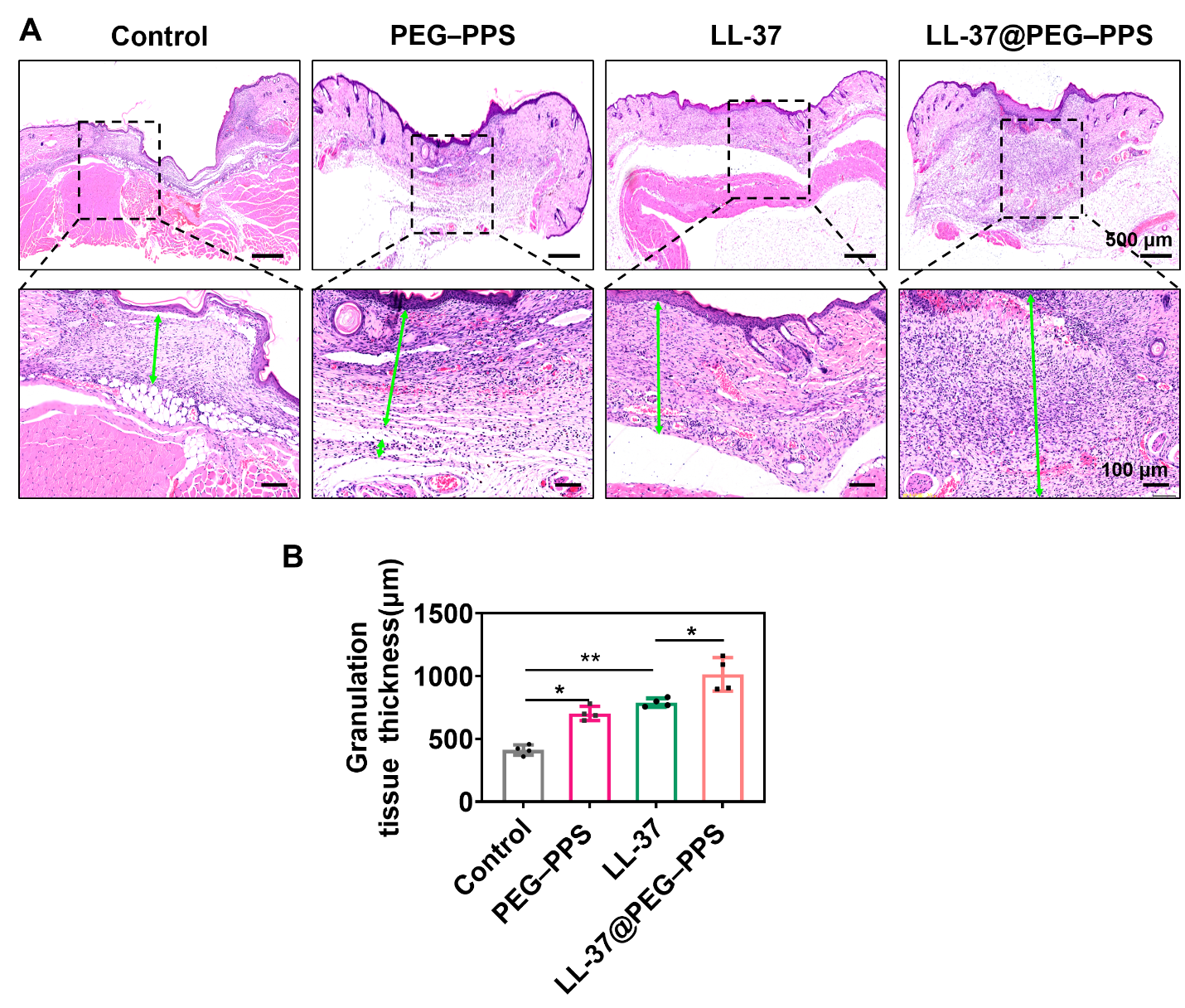


**Figure S16.** (A) On the 12th day after injury, representative images of new granulation tissue under different treatment were indicated by the green double arrows. Scale bar: 500 μm and 100 μm, respectively. (B) Statistical analysis of new granulation tissue under different treatment on the 12th day after injury. Data are represented as mean ± SD (n = 4). **p* < 0.05, ***p* < 0.01, n.s., no significance, (one-way ANOVA).


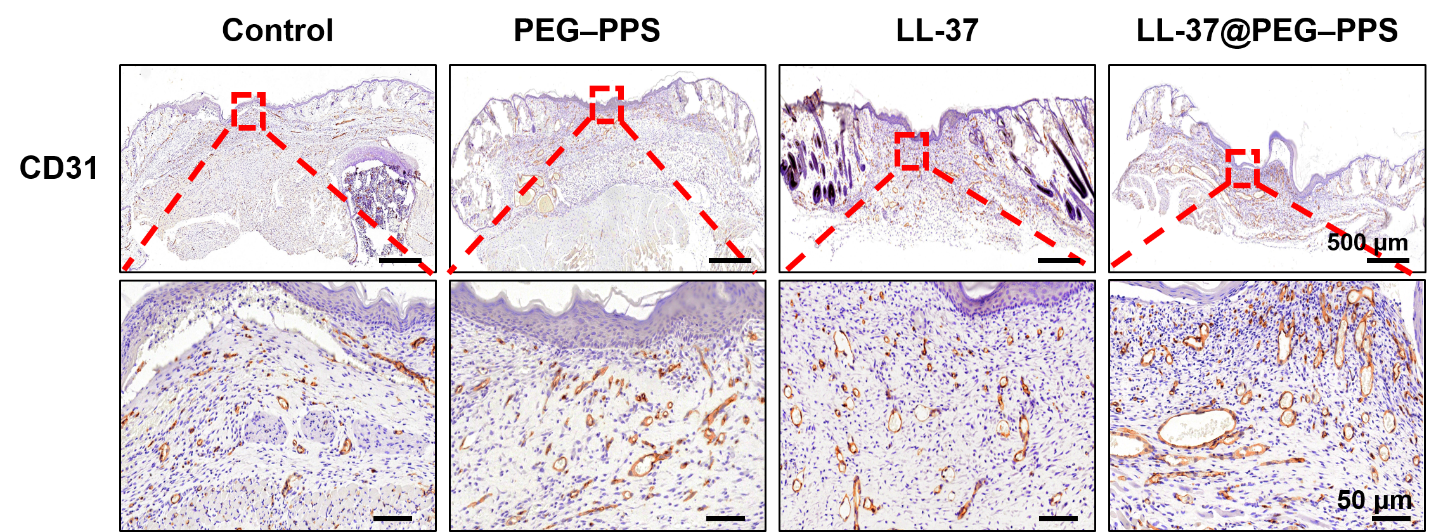


**Figure S17.** CD31 IHC staining of diabetic wound tissues of each group (scale bar: 500 μm and 50 μm).


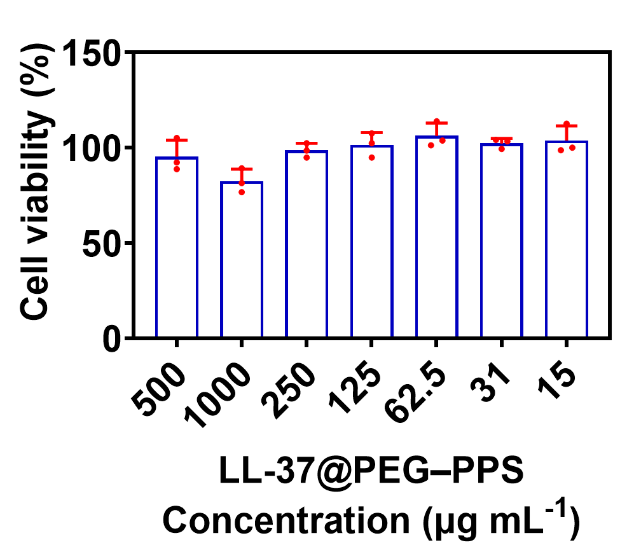


**Figure S18.** The viability of HUVEC cells treated with different concentrations of LL-37@PEG–PPS after 24 h (n = 3).

**Report S1. BLISS model analysis of the synergistic effect of LL-37 and PEG–PPS in promoting wound healing.**


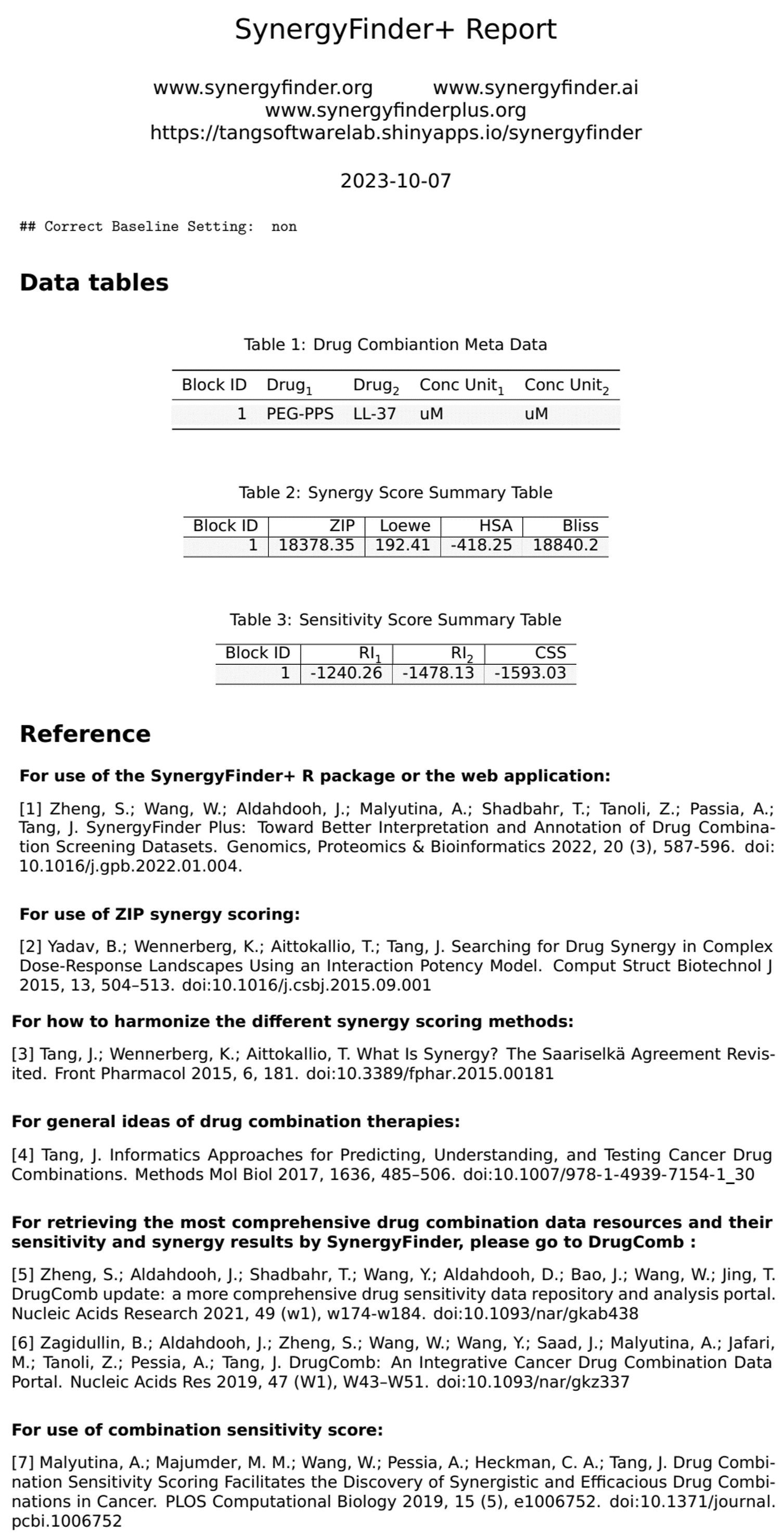

Supplement: Supplementary file 1 — Data S1. Supporting Information. [file BTM2-9-e10619-s001.docx]
